# Differential gene expression during early development in recently evolved and sympatric Arctic charr morphs

**DOI:** 10.7717/peerj.4345

**Published:** 2018-02-07

**Authors:** Jóhannes Guðbrandsson, Sigríður Rut Franzdóttir, Bjarni Kristófer Kristjánsson, Ehsan Pashay Ahi, Valerie Helene Maier, Kalina Hristova Kapralova, Sigurður Sveinn Snorrason, Zophonías Oddur Jónsson, Arnar Pálsson

**Affiliations:** 1 Institute of Life and Environmental Sciences, University of Iceland, Reykjavík, Iceland; 2 Freshwater Division, Marine and Freshwater Research Institute, Reykjavík, Iceland; 3 Biomedical Center, University of Iceland, Reykjavík, Iceland; 4 Hólar University College, Sauðárkrókur, Iceland; 5 Karl-Franzens-Universität, Graz, Austria

**Keywords:** Divergence, Salmonid, Transcriptome, Evolution, Lake Thingvallavatn, *Salvelinus alpinus*, 3′-bias, RNA sequencing

## Abstract

Phenotypic differences between closely related taxa or populations can arise through genetic variation or be environmentally induced, leading to altered transcription of genes during development. Comparative developmental studies of closely related species or variable populations within species can help to elucidate the molecular mechanisms related to evolutionary divergence and speciation. Studies of Arctic charr (*Salvelinus alpinus*) and related salmonids have revealed considerable phenotypic variation among populations and in Arctic charr many cases of extensive variation within lakes (resource polymorphism) have been recorded. One example is the four Arctic charr morphs in the ∼10,000 year old Lake Thingvallavatn, which differ in numerous morphological and life history traits. We set out to investigate the molecular and developmental roots of this polymorphism by studying gene expression in embryos of three of the morphs reared in a common garden set-up. We performed RNA-sequencing,* de-novo* transcriptome assembly and compared gene expression among morphs during an important timeframe in early development, i.e., preceding the formation of key trophic structures. Expectedly, developmental time was the predominant explanatory variable. As the data were affected by some form of RNA-degradation even though all samples passed quality control testing, an estimate of 3′-bias was the second most common explanatory variable. Importantly, morph, both as an independent variable and as interaction with developmental time, affected the expression of numerous transcripts. Transcripts with morph effect, separated the three morphs at the expression level, with the two benthic morphs being more similar. However, Gene Ontology analyses did not reveal clear functional enrichment of transcripts between groups. Verification via qPCR confirmed differential expression of several genes between the morphs, including regulatory genes such as *AT-Rich Interaction Domain 4A (arid4a)* and *translin (tsn)*. The data are consistent with a scenario where genetic divergence has contributed to differential expression of multiple genes and systems during early development of these sympatric Arctic charr morphs.

## Introduction

Phenotypic diversity provides the raw material for evolution and is influenced by variation in gene expression during development and the lifespan of individuals. Variation in gene expression is both influenced by genetics (Jin et al., 2001; Oleksiak, Churchill & Crawford, 2002) and environmental factors (Giger et al., 2006; Danzmann et al., 2016). Gene expression can change because of neutral evolution, as well as positive and purifying selection (Romero, Ruvinsky & Gilad, 2012). In the context of development the combined effects of purifying or stabilizing selection on existing traits and genetic drift, may lead to developmental system drift (True & Haag, 2001), that is alterations in gene expression and the functions of developmental circuits. Analyses of gene expression in developing organisms can reveal variation in the developmental circuits and the phenotypes they influence (Garfield et al., 2013) and alterations in the parameters of these networks (Ludwig et al., 2005). Evolutionary developmental biology seeks answers to questions like which developmental and cellular systems influence variation in adaptive traits and are some developmental processes, time points or tissues more prone/amenable to natural selection than others (Kopp, Duncan & Carroll, 2000; Carroll, 2008; Stern & Orgogozo, 2008)?

To address questions about the interplay of natural selection, developmental biology and drift in evolutionary divergence, we can study the developmental and molecular basis of natural diversity in recently diverged species or diverging populations within species. For example, studies of the Galapagos finches (*Geospiza* spp.) revealed that expression of *bone morphogenetic protein 4* and *calmodulin* during beak development has strong effects on beak depth and width (Abzhanov et al., 2004; Abzhanov et al., 2006), which are important characteristics for fitness (Grant, 1999; Grant & Grant, 2008). At the population level it was found that differential expression of the *Agouti* gene in hair follicles in deer mice (*Peromyscus* spp.) correlated with differences in coat color which varies among populations (Linnen et al., 2009). Here we set out to study gene expression during early development, in recently diverged populations with profound phenotypic separation, with the broad aim to understand molecular mechanisms related to phenotypic variation and adaptation.

### Polymorphic and sympatric Arctic charr *Salvelinus alpinus* as a model to study evolution

After the last glaciation (∼12,000 years ago) salmonid species and threespined sticklebacks (*Gasterosteus aculeatus*) were prominent among fish species that colonized newly formed lakes and rivers of the northern hemisphere (Wootton, 1984; Noakes, 2008; Klemetsen, 2010).

Several fish species of northern freshwaters have diverged locally to form polymorphic systems, usually related to utilization of different resources (resource polymorphism, Skúlason & Smith, 1995; Smith & Skúlason, 1996; see additional refs. in Snorrason & Skúlason, 2004). This is seen in many salmonids (Robinson & Parsons, 2002; Muir et al., 2016) and in Arctic charr many cases of phenotypically distinct sympatric morphs have been reported in post glacial lakes, for instance in Norway, Scotland and Iceland (Telnes & Sægrov, 2004; Adams et al., 2007; Klemetsen, 2010). In Iceland, Arctic charr is found as anadromous or non-anadromous resident populations in rivers and lakes. Many of the resident populations have become landlocked. The anadromous charr usually grow large and have pointed snouts with a terminal mouth resembling limnetic morphology. Many landlocked populations differ in feeding morphology, some feed on zooplankton or fish (limnetic morphs) while others utilize benthic prey (benthic morphs, Skúlason et al., 1992), as is common in northern polymorphic freshwater fish species (Bernatchez et al., 2010). Although somewhat variable in morphology, benthic charr are distinct from limnetic charr, with typically darker body, blunt snout and sub-terminal mouth. In Iceland they are most commonly found as dwarf morphs (adult length less than 15 cm) in isolated spring habitats in the neo-volcanic zone (Kristjánsson et al., 2012). Population genetics suggest that these benthic dwarfs have evolved repeatedly in groundwater springs across the island (Kapralova et al., 2011). Larger benthic forms do exist, with similar phenotypic characters as the dwarfs but larger adult size (Skúlason et al., 1992; Kristjánsson et al., 2011).

**Figure 1:**
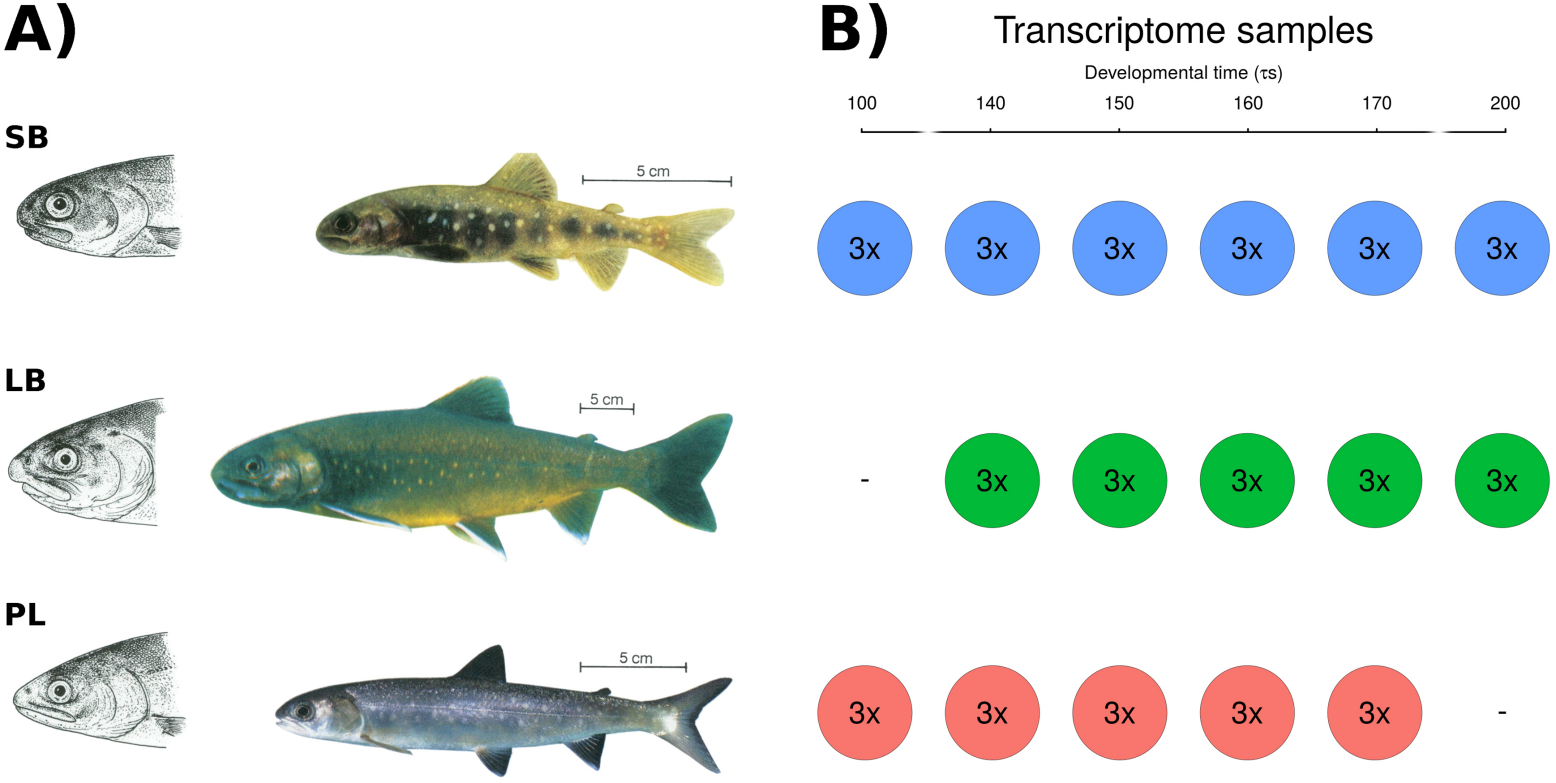
The phenotypically distinct sympatric Arctic charr and the experimental set-up. (A) Four sympatric morphs inhabit Lake Thingvallavatn, three of which are studied and pictured here: small benthic (SB), large benthic (LB) and planktivorous (PL) charr. They differ in size (size bars = 5 cm), the shape of the head and jaws (see drawings) and pigmentation. Adapted from Sandlund et al. (1992), ©Wiley-Blackwell, drawings by Eggert Pétursson. (B) Embryos from pure crosses of the three morphs were sampled at six developmental timepoints prior to hatching, from 100 *τs* to 200 *τs* (circles) for RNA sequencing. During this period of development somatogenesis is complete and gill arches, jaws and many other structures are forming (Fig S1). Three biological replicate samples (3 ×) were taken for each morph and developmental timepoint, each sample being a pool of mRNA from three embryos. Six timepoints were sampled in SB-charr, and five in LB- and PL-charr. In total there were 48 samples, composed of 144 individual charr embryos. The coloring scheme indicating morphs (blue: SB, green: LB, red: PL) will be retained throughout the manuscript.

Sympatric Arctic charr morphs, found in several lakes, most often separate into benthic or limnetic morphotypes varying in many traits (morphology, behavior, color, life history characteristics, habitat use) (Snorrason & Skúlason, 2004). A well studied example of polymorphic Arctic charr are the four charr morphs of Lake Thingvallavatn (Fig. 1A). They differ distinctly in various traits, e.g., adult size, age at maturity, head and body morphology, coloration, behavior and habitat use (Sandlund et al., 1992). In the lake there are two limnetic morphs, the smaller planktivorous morph (PL, 15–25 cm adult length) that feeds on zooplankton, and the larger piscivorous morph (PI, 25–60 cm adult length) that mainly feeds on threespined stickleback (Snorrason et al., 1989; Malmquist et al., 1992). The lake harbors two benthic morphs, small benthic charr (SB, 12–20 cm adult length) and large benthic charr (LB, 25–60 cm adult length) both feeding on bottom-dwelling invertebrates in the lava substrate habitat along the shores (Sandlund et al., 1992). Rearing experiments showed that morphological and behavioral differences among the morphs arise early in development (Skúlason et al., 1993; Skúlason et al., 1996), and subsequent studies of developing embryos and juveniles showed significant differences in cartilage and bone formation (Eiriksson, Skulason & Snorrason, 1999; Eiriksson, 1999). Recently Ahi et al. (2014) used geometric morphometrics to capture variation in craniofacial structures among progeny of three of the morphs (PL-, LB- and SB-charr) soon after hatching (280 − 285*τs*, see Materials and Methods for explanation of relative age measured in *τs*). For the ventral shape of the lower jaw and hyoid arch, distinct differences between the morphs were found at 305*τs*, (Ahi et al., 2014). Experiments corroborate the contribution of genetic differences, but also demonstrated significant plastic potential of these morphs. The phenotypic plasticity of Arctic charr, and related salmonids is well documented (Nordeng, 1983; Hindar & Jonsson, 1993; Skúlason, Snorrasson & Jónsson, 1999). Studies on developing charr have revealed plastic responses to environmental factors like temperature, water velocity and food type (Adams & Huntingford, 2004; Grünbaum et al., 2007; Jonsson & Jonsson, 2014). Studies of limnetic and benthic charr morphs in Iceland show food type can affect growth and the shape of the feeding apparatus in early feeding juveniles (Parsons, Skúlason & Ferguson, 2010; Parsons et al., 2011; Küttner et al., 2013). Furthermore, egg volume, which varies considerably within and among females, is positively correlated to yolk depletion rate and fork length at hatching and at first feeding in aquaculture charr (Leblanc, Kristjánsson & Skúlason, 2016). Here we study gene expression during the early development of sympatric morphs, reared in a common garden that reduces the influence of environmental variations. Note however, the experimental design can not distinguish between genetic and parental effects on embryonic gene expression.

### Genetic variation in polymorphic and sympatric Arctic charr

The earliest population genetic studies found little genetic separation of the sympatric morphs in Lake Thingvallavatn (Magnusson & Ferguson, 1987; Danzmann et al., 1991; Volpe & Ferguson, 1996). The first microsatellite study detected subtle differences (Gíslason, 1998) and the second study with 10 markers estimated overall *F*_*ST*_’s = 0.039, between the LB-, SB- and PL-charr (Kapralova et al., 2011). More recently, we detected *F*_*ST*_’s larger than 0.25 between morphs for variants in two immunological genes (Kapralova et al., 2013) and a few other loci (Guðbrandsson et al., 2016), suggesting substantial genetic separation at specific loci among those sympatric charr morphs. There is a need to study underlying developmental mechanisms, e.g., how differential expression or function of genes promotes differences in charr development and phenotypes. To date, few studies have addressed these issues. The candidate gene approach illustrates how embryonic morphogenetic mechanisms may influence phenotypic diversity and speciation (Abzhanov et al., 2004; Abouheif et al., 2014). A qPCR study on muscle tissues in charr, showed that expression of three genes in the *mTOR*-pathway distinguishes five small benthic morphs from two limnetic morphs in Iceland (Macqueen et al., 2011). On the other hand the myogenic *paired box protein 7 (Pax7)* gene was not differentially expressed during development in Lake Thingvallavatn morphs (Sibthorpe et al., 2006).

Genome wide methods are the new norm, for example population genomics (Pease et al., 2016) or transcriptome screens (Perry et al., 2012) to investigate patterns of divergence or loci of adaptation. In this context it is worth stressing that salmonids, due to the fourth whole genome duplication of the linage (Ss4R) 88–103 million years ago (Moghadam, Ferguson & Danzmann, 2011; Macqueen & Johnston, 2014; Berthelot et al., 2014; Lien et al., 2016), have quite complex genomes. The extra paralogs and chromosomal changes (Macqueen & Johnston, 2014; Nugent et al., 2017) complicate genome and transcriptome assemblies and analyses (Norman, Ferguson & Danzmann, 2014; Lien et al., 2016). To date the genome of two salmonids, Atlantic salmon (*Salmo salar*, hereafter salmon) (Lien et al., 2016) and rainbow trout (*Oncorhynchus mykiss*) (Berthelot et al., 2014), have been sequenced and annotated, but comparable resources are not available for Arctic charr.

We are interested in elucidating the developmental and molecular basis of trophic diversity in Arctic charr. Previously we deployed high throughput sequencing on embryos of SB-charr from Lake Thingvallavatn and an Icelandic aquaculture-charr breeding strain, to identify expression differences in microRNA and protein coding genes (Kapralova et al., 2014; Guðbrandsson et al., 2016). The miRNA sequencing revealed differential expression in 72 microRNAs, including some related to development of the brain and sensory epithelia, skeletogenesis and myogenesis (Kapralova et al., 2014). Similarly, the mRNA transcriptome (Guðbrandsson et al., 2016) indicated differences in the function of several pathways and genes, including metabolic, structural and regulatory genes. In that study we hypothesized that the observed expression divergence in mitochondrial functions (Guðbrandsson et al., 2016) reflected either strong artificial selection for growth rate in aquaculture-charr or altered life history of SB-charr in Lake Thingvallavatn by selection for early maturation with the trade-off in energy allocation highly favouring the production of gonads rather than body growth (Jonsson et al., 1988). Based on the transcriptome data from Guðbrandsson et al. (2016) and known craniofacial expression in other species we chose candidate genes to analyze gene expression with qPCR in limnetic and benthic morphs. Briefly, the data showed that a number of genes with conserved co-expression, most of which are involved in extracellular matrix organization and skeletogenesis (and *ETS proto-oncogene 2, transcription factor, Ets2*), differed in expression between benthic and limnetic morphs (Ahi et al., 2013; Ahi et al., 2014). Furthermore, employing the candidate gene approach on preliminary analysis of the data presented here, linked the Aryl-hydrocarbon receptor pathway to benthic-limnetic divergence in charr (Ahi et al., 2015).

Here we study the early developmental transcriptome of three of the four sympatric morphs from Lake Thingvallavatn (LB-, SB- and PL-charr) with the aim of identifying genes and molecular systems that have featured in the divergence of the Thingvallavatn morphs. The expression divergence can also shed light on the evolutionary relationship of the three morphs under study. Our previous developmental RNA-sequencing study of Arctic charr (Guðbrandsson et al., 2016) provided a useful start off for analysing gene expression and developmental pathways associated with the benthic vs. limnetic differences (Ahi et al., 2014; Ahi et al., 2015). The study described here differs from Guðbrandsson et al. (2016) in several aspects: (i) it focuses on an earlier window of development in higher temporal resolution (six time points at 100–200 *τs* vs four from 141–433 *τs*). This window of development precedes and covers the formation of key craniofacial structures, e.g., those required for feeding apparatus functions (gill arches and elements of the jaws) leading up to 200 *τs* when most of the viscerocranium is in place (Fig S1) (Kapralova et al., 2015). The developmental pathways related to these structures lay the ground well before they become visible. (ii) The present study compares expression in three Thingvallavatn morphs whereas in the previous study the comparison was between Thingvallavatn SB-charr and an aquaculture stock of mixed origin, which has a typical limnetic-like head morphology but has been subjected to strong artificial selection for growth. (iii) Because of the high coverage and length of the reads in the current study (101 bp, paired-end) we were able to perform *de-novo* transcriptome assembly, which was not possible with the short (36 bp) reads of the previous study. We were therefore able to map reads onto a charr transcriptome instead of making use of *S. salar* EST’s.

Based on the documented differences in jaw morphology soon after hatching (Ahi et al., 2014), we anticipated substantial expression differences in systems related to growth and development of craniofacial structures. However as RNA was isolated from whole embryos, we also expect differences in genes related to physiological systems and development of other body parts. As expected, the data reveal substantial changes in gene expression during early development and importantly also morph specific expression differences in a large number of transcripts. In sum, multiple genes in many pathways were found to be differentially expressed in early development of these recently evolved sympatric charr morphs. The data set the stage for detection of genetic and environmental underpinnings of the observed phenotypic and developmental differences between the morphs.

## Materials and Methods

### Sampling, rearing and developmental series

Embryos from crosses of wild caught fish were reared in a common garden environment (see below) at Hólar University College aquaculture facility in Verið (Sauðárkrókur, Iceland) as in previous studies (Ahi et al., 2013; Guðbrandsson et al., 2016). Embryos from three morphs from Lake Thingvallavatn were studied (Fig. 1).

Parents were fished in Lake Thingvallvatn with the permissions both from the owner of the land in Mjóanes and from the Thingvellir National Park commission. Ethics committee approval is not needed for regular or scientific fishing in Iceland (The Icelandic law on Animal protection, Law 15/1994, last updated with Law 55/2013).

Embryos were reared at ∼5 °C with constant water flow and in complete darkness. As the morphs spawn at different times, slight fluctuations in water temperature could not be avoided. Water temperature was recorded twice daily and the average was used to estimate the relative age (*RA*) of the embryos using *τ*-somite units (*τs*) (Gorodilov, 1996). The following formula was used to calculate the relative age (*RA*) at days post fertilization (*n*) using the average daily temperature (*t*_*i*_).

\begin{eqnarray*}R{A}_{n}=\sum _{i=1}^{n}1440\cdot (1/1{0}^{3.0984-0.0967{t}_{i}+0.00207{t}_{i}^{2}}) \end{eqnarray*}

Sampling of embryos for RNA extraction was performed by Holar University College aquaculture Research Station (HUC-ARC) personnel. Embryos were sampled at designated timepoints, placed in RNAlater (Ambion), stored at +4 °C overnight and frozen at −20°C. HUC-ARC has an operational license according to Icelandic law on aquaculture (Law 71/2008), which includes clauses of best practices for animal care and experiments.

Embryos from pure multi-parent crosses of the three morphs were sampled at six developmental timepoints prior to hatching (see below and Fig S1), from 100 *τs* to 200 *τs* for RNA sequencing (circles in Fig. 1). Three biological replicate samples (3 ×) were taken for each morph and developmental timepoint, each sample containing three embryos, where each embryo came from the same cross. Six timepoints were sampled in the SB-charr, and five in the LB- and PL-charr. Hence in total 48 samples were sequenced, composed of 144 individual charr embryos.

Most of the samples came from offspring of crosses created in the 2010 spawning season (SB 150–200 *τs*, PL 140–170 *τs*, LB 140–200 *τs*). For SB- and PL-charr, eggs from 10 females were pooled and fertilized with milt from 10 males from the same morph. For LB-charr the same setup was used except that five females and five males were used. Because of laboratory failure (samples destroyed), we had to replace three morph and timepoint combinations. For 100*τs* in PL-charr, we used samples from the 2011 spawning season (generated with the identical crossing setup). Similarly, SB-charr samples from timepoints 100 and 140 *τs* were replaced with material from two single parent crosses generated 2011. Samples SB100A and SB100B came from the one cross but sample SB100C and all samples for timepoints 140 *τs* were from the second cross. The samples from 2011 did not show aberration from other samples in principal component analyses (PCA) of the expression data (Fig S2). For qPCR two timepoints (150 *τs* and 170 *τs*) were sampled for all three morphs with the same setup, all from crosses made in 2010.

### Staining of embryos for developmental series

Samples of LB-charr embryos from all timepoints were fixed in 4% PFA. Samples from 140–200 *τs* were stained for cartilage (alcian blue) and bone (alizarin red) using a modified protocol from Walker & Kimmel (2007). All samples were stained simultaneously. Stained individuals were placed in a petri dish containing 50 ml of 1% agarose gel and immobilized with insect needles to ensure the correct positioning of the embryo. The head of each individual was photographed ventrally using a Leica (MZ10) stereomicroscope. Between 140 *τs* and 200 *τs* major craniofacial elements appear as clear units of cartilage for example at 150 *τs* the formation in the ventral aspect of the two trabeculae, the Meckel’s cartilages and palatoquatrates can be observed, shortly followed by the emergence of major elements of the hyoid and branchial arches (160–170 *τs*) (Fig. S1B). The minor elements (the hypo- and basi-branchials) of these arches start to appear later (200 *τs*) (Fig. S1B). The ethmoid plate starts forming around 180 *τs* and is almost fully fused centrally at 200 *τs*. Rudiments of the maxillae can be seen as early as 200 *τs*.

### RNA extraction and transcriptome sequencing

For RNA extraction embryos were dechorionated and homogenized with a disposable Pellet Pestle Cordless Motor tissue grinder (Kimble Kontes, Vineland, NJ, USA) and RNA was extracted using TRIzol reagent (Thermo Fisher, Waltham, MA, USA) according to the manufacturers instructions. RNA quantity was examined using a NanoDrop ND1000 (Labtech, East Sussex, UK) spectrophotometer. An Agilent 2100 Bioanalyzer (Agilent Technologies, Santa Clara, CA, USA) was used to assess RNA quality and samples with high RNA integrity number (RIN, an estimate of RNA quality, Schroeder et al., 2006) were selected. Only four samples had RIN below 9 (Table S1). Sequencing libraries were prepared using the TruSeq RNA Sample Preparation kit (Illumina, San Diego, CA, USA) according to the manufacturer’s protocol (Release 15008136, November 2010). mRNA was purified on oligo-(dT) attached magnetic beads, eluted and fragmented at 94 °C for 2 min, to generate fragments of c.a. 130–290 bases. First strand cDNA synthesis was performed using random hexamer primers, followed by RNase treatment and second strand synthesis. The cDNA ends were repaired and adenylated before the ligation of indexing adapters. The libraries were PCR amplified (15 cycles). Samples were quantified with NanoDrop and quality estimated with BioAnalyzer before they were pooled and sequenced on Hiseq 2000 at deCODE genetics (Reykjavik, Iceland), yielding 101 bp paired-end reads. The raw reads were deposited into the NCBI SRA archive under BioProject identifier PRJNA391695 and with accession numbers: SRS2316381 to SRS2316428.

### Assembly, abundance estimation and annotation

The sequencing reads were quality trimmed and adapters removed using Trim Galore (version 0.3.3, Krueger, 2012) before assembly. Bases with Phred-quality below 10 were trimmed off. Reads that were less than 20 bp after trimming were removed and the mate of the read was also removed from downstream analysis. The quality filtered reads from all samples were assembled using Trinity (version v2.1.0, Grabherr et al., 2011) with the default parameters, except the “min_kmer_cov” was set to two to reduce memory use. Preliminary analysis using salmon EST contigs (Di Génova et al., 2011) as reference indicated extensive RNA degradation and subsequent 3′ bias in all samples for one timepoint (160 *τs*) in two (LB and PL) out of the three morphs. This timepoint was thus excluded from gene expression analyses as 3′ bias can have drastic effects on expression estimations (Sigurgeirsson, Emanuelsson & Lundeberg, 2014). RNA degradation also affected other samples, see below. We used Kallisto (version v0.42.4, Bray et al., 2016) to estimate the abundance of transcripts. Kallisto was run with default parameters and 30 rounds of bootstrapping. Only transcripts with more than 200 estimated reads total in the samples, were retained for annotation and expression analysis.

The transcripts were annotated using the Trinotate pipeline (version 2.0.2, Haas, 2015). Trinotate runs the assembled contigs through a few programs for detecting coding sequences, protein structures and rRNA genes as well as running blast on SwissProt and TrEMBL databases for ortholog detection (see http://trinotate.github.io/). Trinotate was run with the default parameters except that we set the *E*-value cutoff for blast searches to 10^−20^. If two or more open reading frames (ORFs) were predicted for a transcript we excluded ORFs that did not blast to the trEMBL database. If ORFs from the same transcript overlapped we excluded the one with higher *E*-value.

Orthologs of the transcripts in salmon and rainbow trout mRNA and protein sequences were found using blastn and blastx respectively. The annotations for the rainbow trout genome were obtained from Berthelot et al. (2014), (http://www.genoscope.cns.fr/trout/data/, version from 2014-05-19). The annotation for the salmon genome came from two different sources; NCBI *Salmo salar* Annotation Release 100 (https://www.ncbi.nlm.nih.gov/genome/annotation_euk/Salmo_salar/100/, retrieved 2015-12-17) and SalmoBase (Samy et al., 2017; http://salmobase.org/), version from 2015-09-18. For each reference dataset we only retained the best match for each transcript. We set the *E*-value cutoff for blastn searches to 10^−50^, minimum percent identity to 85% and the transcript was required to cover at least 50% of the reference transcript. For blastx searches we set the *E*-value cutoff to 10^−20^, minimum percent identity to 75% and mandated that the transcript should cover at least 20% of the reference protein. Scripts from the Trinity suite (Grabherr et al., 2011) were used to group discontinuous alignments and calculate the alignment coverage of reference transcripts.

### Estimation of RNA degradation and 3′-bias

To estimate read coverage across the length of transcripts we supplied pseudobam files from Kallisto to eXpress (version 1.5.1; Roberts & Pachter, 2012). eXpress uses an expectation-maximization (EM) algorithm for read placement based on sequence composition and transcript expression. We used the default parameters except for the ‘batch’ option which was set to 10 to get more EM-rounds. To estimate 3′-bias we chose 381 long transcripts with high read coverage and which spanned almost full length genes. In more detail, the transcripts were chosen if at least 90% of their sequence aligned to over 90% of a salmon transcript (based on SalmoBase annotation). We restricted the analysis to transcripts between 2,000 and 6,000 bp in length, with high read coverage and little variation between samples. The coverage was estimated in 100 bins over the length of each transcript. The 3′-bias was estimated as a percentage of coverage for the 3′half of each transcript compared to the total transcript, and the average for all of 381 transcript calculated for each of the 48 samples. The calculations were performed in the R environment (R Core Team, 2017). This quantity will be referred to as 3′ coverage hereafter and used as an estimate of 3′-bias for each sample.

### Estimating expression differences among morphs

Kallisto (Bray et al., 2016) was used to estimate transcripts abundance per sample. Transcripts with at least 200 mapped reads were subjected to expression analysis, using the R-package Sleuth (Pimentel et al., 2017) to fit linear models. The full model (FM) included morph (*M*) and developmental time (*T*) and the interaction of morph and developmental time (*M* × *T*). We also fitted three reduced models excluding different factors of the full model to test for influences of that factor. In addition we took the 3′ coverage (described above, *z* in formulas below) into account. We fitted the 3′ coverage as a second degree polynomial to allow the effect on expression to be non-linear while keeping the model as parsimonious as possible. We compared the full model to model R1 to test for the interaction term or morph effect within time-points. We compared R1 to R2 to test for overall morph effect and finally we compared R1 to R3 to check for influences of developmental time on gene expression. The models were compared with a likelihood ratio test to check for significance of variables.

(FM) \begin{eqnarray*}{y}_{ijk}& ={M}_{i}+{T}_{j}+(M\times T)_{ij}+{\beta }_{1}{z}_{k}+{\beta }_{2}{z}_{k}^{2}\end{eqnarray*}

(R1) \begin{eqnarray*}{y}_{ijk}& ={M}_{i}+{T}_{j}+{\beta }_{1}{z}_{k}+{\beta }_{2}{z}_{k}^{2}\end{eqnarray*}

(R2) \begin{eqnarray*}{y}_{ijk}& ={T}_{j}+{\beta }_{1}{z}_{k}+{\beta }_{2}{z}_{k}^{2}\end{eqnarray*}

(R3) \begin{eqnarray*}{y}_{ijk}& ={M}_{i}+{\beta }_{1}{z}_{k}+{\beta }_{2}{z}_{k}^{2}.\end{eqnarray*}

To gauge the effect of including 3′ coverage as an explanatory variable, we also ran models excluding 3′ coverage. We tested if 3′-bias had an effect on expression (model FM vs R4). We also tested for interaction, morph and time effect without taking 3′-bias into account (R4 vs R5, R5 vs R6 and R5 vs R7).

(R4) \begin{eqnarray*}{y}_{ijk}& ={M}_{i}+{T}_{j}+(M\times T)_{ij}\end{eqnarray*}

(R5) \begin{eqnarray*}{y}_{ijk}& ={M}_{i}+{T}_{j}\end{eqnarray*}

(R6) \begin{eqnarray*}{y}_{ijk}& ={T}_{j}\end{eqnarray*}

(R7) \begin{eqnarray*}{y}_{ijk}& ={M}_{i}.\end{eqnarray*}

Sleuth uses false discovery rate (*fdr*) to adjust for multiple testing (Benjamini & Hochberg, 1995). Transcripts with significant morph/time interaction or morph effect (*fdr* < 0.01) were classified into 16 clusters using the Mfuzz-package (Futschik, 2015). For clustering we used log-transformed estimates of transcripts per million (tpm) normalized by 3′-bias, with the fuzzification parmeter (*m*) set to 1.1. To visualize the differences between morphs we performed principle component analysis (PCA) in R on the expression estimates, only for transcripts in clusters with morph effects and time-invariant expression differences between morphs (clusters A–E).

The goseq-package in R (Young et al., 2010) was used to test for enrichment of Gene Ontology (GO) categories of biological processes within each cluster. The annotation from SalmoBase was used and transcripts were also mapped to all the ancestors of annotated GO categories using the GO.db-package in R (version 3.2.2; Carlson, 2015). To get an overall signal and increase statistical power, rather than trying to get a specific signal from incompletely annotated data, we decided to focus on GO-categories at specific positions in the GO-category relationship tree. For enrichment tests we used only categories with the longest path to the root of the GO-tree at least three steps and the shortest path to root no longer than four steps. Note that different paths from a specific category to root can be of different lengths. For each cluster we ran two enrichment tests. First on the transcript level where length bias was taken into account (Young et al., 2010). Second we ran enrichment test for salmon genes (based on SalmoBase annotation). A gene was considered to belong to a cluster if a transcript annotated to it belonged to the cluster. For the gene GO-enrichment tests we used a Hypergeometric test without any length correction. A GO-category was only considered significant if significance (*fdr* < 0.01) was found on both transcript and gene level. The gene level was also used to correct for genes with multiple isoforms or incomplete assemblies, which can lead to false positive categories. We clustered significant GO-categories for each cluster using semantic similarity between categories in the zebrafish genome according to the GOSemSim-package in R (Yu et al., 2010) as a distance measurement. The distance matrix for GO-categories was supplied to the hclust function in R and a cutoff of 0.8 was used to categories the GO-categories in to super categories.

### qPCR verification of gene expression

Candidate genes for verification by qPCR were picked based on differential expression between morphs in the transcriptome and in some cases prior data on biological functions. Reference genes to study Arctic charr development have previously been identified (Ahi et al., 2013). Primer3 (Untergasser et al., 2012) was used to design primers (Table S4) and the primers were checked for self-annealing and heterodimers in line with MIQE guidelines (Bustin et al., 2009). Primers for genes with several paralogs were designed for regions conserved among paralogs. RNA extraction followed the same steps as for samples used in the transcriptome. cDNA synthesis followed the same steps as in Ahi et al. (2015): DNA contamination was removed using DNases treatment (New England Biolabs, Ipswich, MA, USA) and cDNA was synthesized with 1 µg of RNA using the High Capacity dDNA RT kit (Applied Biosystems, Foster City, CA, USA) in 20 µl reaction volume.

Real-time PCR was performed in 96 well-PCR plates on an ABI 7500 real-time PCR System (Applied Biosystems, Foster City, CA, USA). The normalized relative expression of genes in whole embryos was estimated from the geometric mean expression of two reference genes, *β-actin (actb)* and *ubiquitin-conjugating enzyme E2 L3 (ub2l3)*. To visualize differences among morphs and time, the normalized expression was presented as relative to the expression of one of three samples in PL at 150 *τs* (calibration sample). Relative expression was calculated using the 2^−ΔΔ*Ct*^ method of Livak & Schmittgen (2001). Statistical analysis was performed using the Δ*C*_*T*_-values with a two-way ANOVA with GLM function in R.

\begin{eqnarray*}{y}_{ijk}={M}_{i}+{T}_{j}+(M\times T)_{ij}+{\varepsilon }_{ijk}. \end{eqnarray*}

The residuals were normally distributed for all data. Genes with significant morph effect was followed up on by performing Tukey’s post-hoc test, on relative expression ratios (Δ*C*_*T*_s).

## Results and discussion

### Transcriptome sequencing, assembly and annotation

The number of sequenced paired-end reads varied among the 48 samples, from 4.5 to 86.9 million. No bias in read number among lanes, indexes, morphs or developmental timepoints was detected, except that timepoint 160 *τs* in LB-charr had low coverage for all three replicates (Table S1). Trinity (Grabherr et al., 2011) *de-novo* assembly yielded 581,474 transcripts which grouped into 449,681 “genes”. After filtering on coverage (minimum of 200 reads aligned) the numbers of transcripts and “genes” decreased to 129,388 and 78,667 respectively. All estimators of length increased with this filtering step, e.g., the N10–N50 statistics (Table 1).

**Table 1:** Summary statistics for the transcriptome assembly, from the raw Trinity output and filtering out transcripts with less than 200 reads mapped. Lengths (in basepairs) of all transcripts and the longest transcript (isoform) for each gene are tabulated.

	Raw	Raw long iso^a^	Filtered	Filtered long iso^a^
Total Trinity ‘genes’	449,681		78,667	
Total Trinity transcripts	581,474		129,388	
GC-content (%)	45.93		47.41	
N10	4,818	3,830	5,858	5,457
N20	3,527	2,417	4,598	4,132
N30	2,685	1,551	3,822	3,317
N40	2,015	1,031	3,218	2,707
N50	1,441	718	2,709	2,197
Median contig length	364	328	1,270	851
Average contig length	757.94	559.54	1,737.01	1,338.73
Total assembled bases	440,720,391	251,613,073	224,748,860	105,235,409

**Notes.**

a Longest isoform for each Trinity gene.

Blastn revealed that the majority of the transcripts had homology with sequences in Atlantic salmon (72% for the NCBI database and 83% for SalmoBase) and rainbow trout (53%). Similar analyses at the protein level (blastx or blastp) found a lower proportion with homology, 43% to 55% depending on the database in the two salmonids and other organisms (Table 2). Even though Arctic charr is considered more closely related to rainbow trout than salmon (Koop et al., 2008; Crête-Lafrenière, Weir & Bernatchez, 2012; Alexandrou et al., 2013) a larger number of transcripts had significant blast hits to salmon. Most likely this reflects the more conservative approach used for annotation of the rainbow trout genome, e.g., requiring genes to have orthology in other vertebrates (Berthelot et al., 2014).

**Table 2:** The number and percent of Trinity transcripts and genes with significant blast hits in different databases, using different blast programs (blastn, blastx and blastp).

Database	Program	Transcripts	Genes	Transcripts (%)	Genes (%)
Ssal NCBI	blastn	93,239	49,281	72.06	62.65
Ssal SalmoBase	blastn	107,068	61,185	82.75	77.78
Omyk	blastn	68,476	33,505	52.92	42.59
Ssal NCBI	blastx	62,548	26,843	48.34	34.12
Ssal SalmoBase	blastx	63,310	27,652	48.93	35.15
Omyk	blastx	55,862	24,533	43.17	31.19
SwissProt	blastx	59,763	24,130	46.19	30.67
TrEMBL	blastx	71,156	30,927	54.99	39.31
SwissProt	blastp	57,702	22,737	44.60	28.90
TrEMBL	blastp	64,442	26,198	49.81	33.30
Total transcripts		129,388	78,667	100	100

We searched reference databases with Arctic charr transcripts, using blastx and blastn, to estimate the number and length of the assembled genes and proteins (Table 3). Hits to 19,122–35,685 proteins were found (depending on database) but with more stringent filters on length (requiring more than 90% coverage) these numbers ranged from 9,367 to 18,593 (Table 3). Using BLAST to align against salmon transcripts (SalmoBase) recovered up to 48,916 hits in the databases (Table 3). Again, more transcripts show homology to salmon than to rainbow trout, which again likely reflects differences in the annotation strategies. We retrieved more hits for transcripts and proteins from the SalmoBase annotation than the Salmon NCBI annotation. The transcripts in the SalmoBase annotation are longer on average compared to the NCBI annotation, therefore our Arctic charr transcripts cover less of each SalmoBase transcripts although more hits are retrieved (Table 3). More than half of the genes covered 90–100% of the predicted protein length, with minimal difference depending on database, while less than half covered more than 90% of the predicted transcript length. This probably reflects the higher divergence between *S. alpinus* and its relatives in the untranslated regions of the transcripts.

**Table 3:** Estimated number of protein coding genes in the *de-novo* assembly. Arctic charr transcripts were compared to different protein databases (using blastx, upper table) and Salmonids mRNA databases (using blastn, lower table). The tables shows the cumulative number of proteins or transcripts covered in each database, ranked by degree of coverage.

	**Proteins**				
Percent covered	TrEMBL	SwissProt	Ssal NCBI	Ssal SalmoBase	Omyk
90–100	15,788	9,367	18,376	18,593	12,829
80–90	18,287	11,610	20,178	20,899	15,070
70–80	20,150	13,163	21,814	23,072	16,476
60–70	21,978	14,404	23,596	25,255	17,713
50–60	23,822	15,484	25,332	27,466	18,885
40–50	25,484	16,478	27,018	29,628	19,850
30–40	26,977	17,380	28,656	31,866	20,718
20–30	28,299	18,219	30,180	33,680	21,353
10–20	29,204	18,907	31,517	35,119	21,775
0–10	29,477	19,122	32,082	35,685	21,888
Total peptides			97,555	195,069	46,585

	**mRNA Transcripts**		
Percent covered	Ssal NCBI	Ssal SalmoBase	Omyk
90–100	12,418	4,189	10,335
80–90	15,671	6,805	13,644
70–80	18,812	9,516	16,182
60–70	22,051	12,820	18,461
50–60	25,748	16,854	20,656
40–50	29,387	21,594	22,113
30–40	33,044	27,691	23,426
20–30	36,822	35,080	24,621
10–20	40,202	43,626	25,671
0–10	41,284	48,916	25,915
Total transcripts	109,584	195,072	46,585

To the best of our knowledge, only two other mRNA-sequencing studies have been conducted on Arctic charr (Norman, Ferguson & Danzmann, 2014; Guðbrandsson et al., 2016). Our previous study of SB-charr and Icelandic aquaculture charr did not involve transcriptome assembly (Guðbrandsson et al., 2016). However Norman, Ferguson & Danzmann (2014) assembled a transcriptome, in their investigation of salinity tolerance in the gills of Canadian aquaculture charr. Their assembly yielded 108,645 assembled contigs, with *N*50 = 2, 588 and around 80% of contigs annotated (using both *S. salar* and *O. mykiss* databases). Our assembly yields fewer “genes” (78,667) after the quality filtering steps, but for downstream analyses we retain more than one transcript per gene. The N50 values of both datasets are similar, but Norman, Ferguson & Danzmann (2014) achieve slightly higher annotation percentage. Our current study provides new data on the transcriptome of Arctic charr from embryos in early development. Integration of these data with genomic sequence data, will be valuable to assemble the complete charr transcriptome and fuel studies of gene gains and losses among salmonid species and populations (Robertson et al., 2017).

### RNA degradation and 3′-bias in the transcriptome

**Figure 2:**
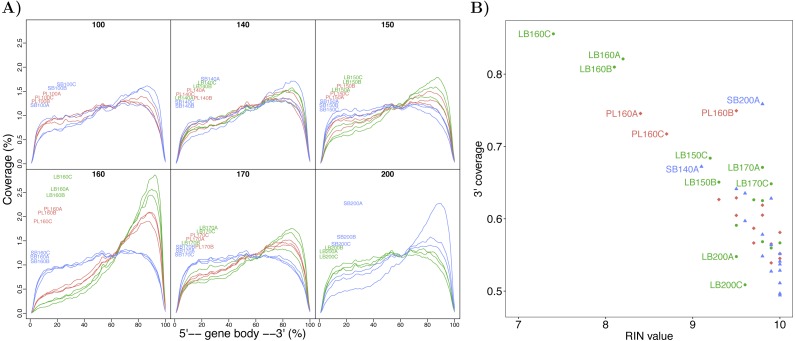
Confounding of 3′-bias with morphs and timepoints in the charr developmental transcriptome data. (A) Average coverage over the length of transcripts for each sample. The coverage was estimated from 381 transcripts that were highly expressed in all samples. The mean coverage for these genes was estimated in 100 windows over the gene body, from the 5′-end to the 3′-end. Samples from different timepoints are graphed separately and colored by morph: LB in green, PL in red and SB in blue. (B) Comparison of RIN-values and 3′ coverage (coverage at the 3′-half divided by total coverage) for each sample, colored by morph.

Preliminary expression analysis with reads mapped to salmon EST’s that showed clear indication of 3′-bias at one timepoint (160 *τs*) led us to remove these samples from the dataset, and take a closer look at position bias. Uneven distribution of reads over transcripts can profoundly influence estimates of expression and subsequent analyses (Wu, Wang & Zhang, 2011). To explore and estimate this bias, we constructed an estimator of 3′ coverage bias and incorporated it into the linear models (see Materials and Methods). The 3′-bias per sample was estimated from 381 nearly full length transcripts in the 2,000–6,000 bp range that had high sequencing coverage in all samples. The patterns of read coverage over the transcripts varied greatly between samples (Fig. 2A). Many samples showed considerable 3′-bias, but more disappointingly the bias was confounded with a variable of chief interest (Morph). The 3′ coverage correlates with the RIN-values of the RNA isolates (Pearson *r* =  − 0.83, *p* = 6.75*e* − 13) but samples with higher 3′ coverage than expected are apparent (e.g., PL160B and SB200A, Fig. 2B). This clearly demonstrates the importance of maintaining high and consistent RNA quality for RNA sequencing if poly-A pulldown is used and the importance of checking for 3′ bias in RNA-seq datasets.

Analyses of differential expression (see below) revealed that the estimated 3′-bias was the second most important factor after developmental time with 32,395 significant transcripts (*α* = 0.01, Table 4). Crucially, the results differed considerably if the 3′-bias term was not included; then more transcripts had significant Morph by Time interaction effect (*M* × *T*) and fewer significant developmental time effect (Time) (Table 4, Fig S3C and Fig S3D). Many transcripts with significant *M* × *T* interaction effect in a model without a 3′-bias term had significant Time effect after normalizing for 3′-bias (Fig S3A). Thus we concluded that involving 3′-bias in the linear models decreased the number of transcripts with potentially false positive *M* × *T* interaction effect.

**Table 4:** Number of differentially expressed transcripts for each effect at different *fdr* cutoffs when taking 3′-bias into account (upper half) and when not taking 3′-bias into account (lower half).

		**With 3′-bias correction**		
*fdr*	3′-bias	*M* × *T*	Morph	Time
<0.05	46,274	14,293	3,381	60,491
<0.01	32,395	8,407	2,002	42,879
<0.001	20,834	3,977	1,075	28,039

	**Without 3′-bias correction**		
*fdr*	*M* × *T*	Morph	Time
<0.05	32,259	2,806	44,710
<0.01	15,789	1,711	27,442
<0.001	4,874	946	16,083

Degradation of RNA is an issue for RNA-sequencing. Particularly poly-A pull-down of degraded mRNA will lead to higher fraction of reads from 3′ end of transcripts (Sigurgeirsson, Emanuelsson & Lundeberg, 2014). Methods for estimating variation in coverage along transcripts, rely on full length sequences (Wu, Wang & Zhang, 2011). Correction for 3′ bias by restricting analyses to 200 bp at the 3′end of transcripts (Sigurgeirsson, Emanuelsson & Lundeberg, 2014) also requires full length sequences or reliable identification of 3′-ends. Neither of those methods were applicable to the current data, as minority of transcripts are of full length, e.g., only 15,671 salmon transcripts in the NCBI database out of 41,284 with homology to Arctic charr are spanned to more than 80% by our contigs (Table 3). Abernathy & Overturf (2016) tested different methods for ribosomal-RNA removal on rainbow trout and concluded that Ribo-Zero (Illumina), which is based on hybridization, gave the best results and should therefore be the method of choice for future studies on incomplete transcriptomes. The use of the estimate of 3′-bias as a covariate reduced the number transcripts with, potentially false, Morph by Time effect. We do not claim that this approach accounts fully for transcript to transcript variation in 3′-bias, so we interpret the following differential expression results cautiously.

### Differential transcript expression between sympatric Arctic charr morphs

**Figure 3:**
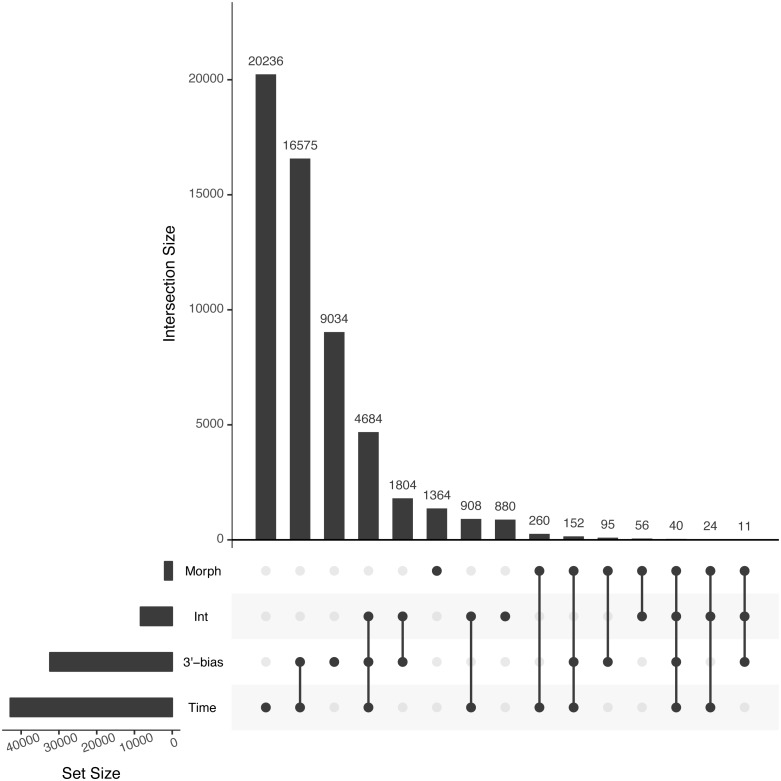
The number of transcripts differentially expressed according to the linear models on developmental timepoint (Time), Morph, 3′-bias and interaction of Morph and Time (int). The set size barplot (sideways) shows cumulated number of transcripts for each of the four main factors, while the intersection size barplot (vertical) shows the number of transcripts significant for each one or a combination of two or more factors. The dots indicate the significant factors or their combinations. For example 20,236 transcripts are only significant for Time effect but 16,575 are significant for both Time and 3′-bias effect.

**Figure 4:**
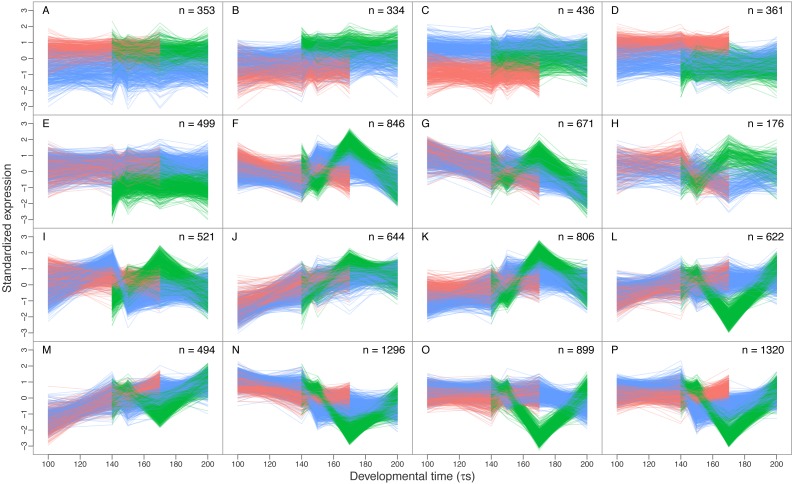
Expression profiles of 16 co-expression clusters. Depicted are transcripts with significant Morph and/or Morph by Time interaction effect, during early charr development (timepoints 100 *τs* to 200 *τs*). Standardized expression normalized by 3′-coverage is plotted against developmental time. Each line is the profile for one transcript. The first five clusters (A–E) capture mainly differences between morphs, while the remaining clusters (F–P) contain almost exclusively transcripts with *MxT* effects (Table 5). The number of transcripts in each cluster is indicated. The morphs are represented by color, SB: blue, LB: green and PL: red.

**Figure 5:**
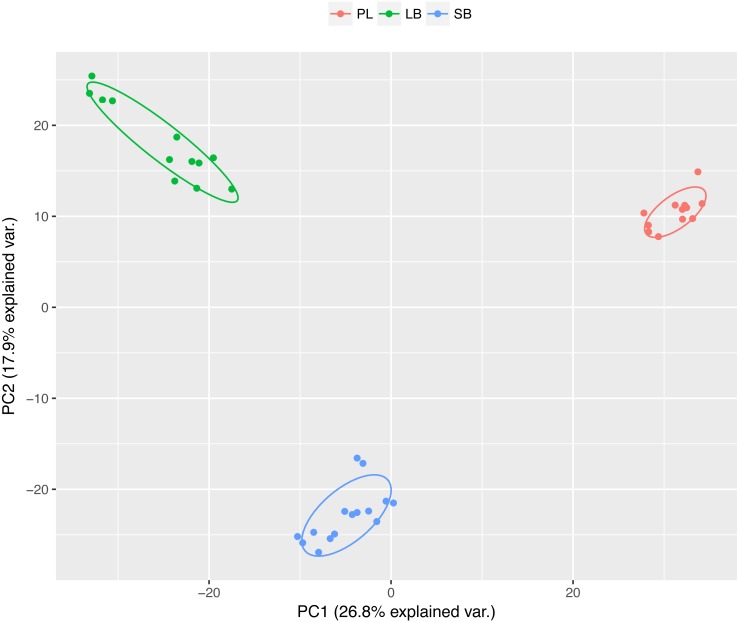
Visualization of the expression differences between the three morphs, with PCA of transcripts in the first five expression clusters (A–E, 1983 transcripts). These clusters were selected as they show an effect of morph, and therefore some separation of morphs in the PCA is expected, but importantly all three morphs are separated from each other. The position of the different morphs in the PCA is informative and indicates that the benthic morphs are more similar in expression as the first axis separates all the morphs with the benthic morphs being close to each other. The second axis separates small benthic from the other two morphs. Standardized expression normalized by 3′-coverage was used as input. Individual samples are graphed (colored by morph, SB: blue, LB: green and PL: red), and overlaid are 68% normal data ellipses for each morph. Figure prepared using the ggbiplot package in R (version 0.55; Vu, 2011).

While developmental time was the most commonly significant factor (42,879 transcripts, Table 4), we were most interested in expression divergence between the three charr morphs. Importantly the 3′-bias correction (above) had limited effect on the number of transcripts with significant overall Morph effect (Fig S3B). We conclude that more than one thousand genes are differentially expressed between developing embryos of the three sympatric morphs. Of the 2,002 transcripts with morph differences (at *fdr* < 0.01), 1,370 were only significant for Morph and no other terms. Further 632 had other terms also significant (some even all), but only 131 transcripts were significant for both Morph and Morph by Time (*M* × *T*) interaction (Fig. 3). A considerably larger number of transcripts (8,407) had a significant *M* × *T* term, with the majority (4,684) also having significant Time and 3′-bias effects. As the 3′-coverage estimator is unlikely to control entirely for the 3′-bias, we suspect the number of transcripts with interaction of Morph by Time may be overestimated. To analyze the differences and changes in the transcripts with Morph and Morph by Time interaction we conducted clustering, yielding 16 co-expression clusters with 176 to 1,320 transcripts each (Fig. 4). Five clusters (A–E) had mostly transcripts with time-invariant Morph effects, but the remaining 11 clusters (F–P) had mainly transcripts with combinations of *M* × *T* and Time effects (Table 5). The data suggest separation between all three morphs at the expression level, for instance in cluster B (334 transcripts). Two of the five Morph effect clusters (C and D) show persistent expression difference between the two benthic (SB and LB) and PL-charr. These contain 797 transcript, while cluster A (with lower expression in SB-charr compared to the other two) has 353 transcripts and 499 were in cluster E (lower expression in LB-charr). To visualize this we performed PCA on the transcripts from these five clusters. This showed all three morphs separate at the transcriptional level (Fig. 5). Some separation of samples based on morph is expected as the genes used for the PCA were selected due to having a significant morph effect, however, importantly in this PCA all three morphs separated completely from each other. Furthermore, the PL-charr separate from the benthic morphs in PC1 (explaining 26.8% of the variance) and the two benthic morphs separate in PC2 (17.9% of the variance).

As transcriptional divergence and genetic divergence tend to be associated (Whitehead & Crawford, 2006), this suggests closer relation of the two benthic morphs, with PL-charr as a more distant relative, consistent with one population genetic study (Volpe & Ferguson, 1996) but incongruent with others (Gíslason, 1998; Kapralova et al., 2011). Preliminary analyses of genetic variation in this transcriptome separates the morphs, and supports closer relation of the benthic morphs (J Guðbrandsson et al., 2018, unpublished data).

We next gauged the functions of the differentially expressed transcripts by Gene Ontology (GO) enrichment analyses, run separately on the 16 co-expression clusters. Note, the GO results should be interpreted cautiously, as mere indications of functional divergence between groups. The analyses were restricted to biological processes and lower level categories. The number of significantly enriched GO categories varied between clusters. Five clusters (A, B, C, E and H) did not have any significant GO enrichment (Table S3), in part reflecting low statistical power as those clusters had the fewest transcripts (176 to 499). The clusters with the largest number of significant GO categories (N, O and P) contained the largest number of transcripts. As was noted above, the five co-expression clusters of transcripts with temporally stable expression that varied between morphs (A–E) had no GO enrichment with the exception of cluster D (transcripts with higher expression in PL-charr, than either LB and SB) which had two GO categories (GO:0097360 and GO:0061450, involved in cell migration and proliferation). Combining all the transcripts in these five clusters in GO-enrichment did not yield any significant GO-categories. The same was true for GO analyses of all transcripts with only Morph effect.

**Table 5:** Number of transcripts differentially expressed (*fdr* < 0.01) for Morph, Time or Morph by Time interaction (*M* × *T*) for co-expression clusters A–E and F–P.

	Clusters	
Effect	A–E	F–P
Morph	1,768	234
Time	307	5,761
*M* × *T*	296	8,111

Just under 700 GO categories were enriched for clusters of genes with significant Morph by Time interactions (Table S3). While dozens up to a hundred GO categories associated with each co-expression cluster, no general pattern emerged. Many different biological processes were enriched in the co-expression clusters, for instance cluster F was enriched for regulation of growth (e.g., GO:0040008) and antigen processing and presentation (e.g., GO:0048002) and cluster L cartilage condensation (e.g., GO:0001502) and limb bud formation (e.g., GO:0060174). A number of categories showed up in three or more clusters, for example; GO:1903047, mitotic cell cycle process (clusters F, G, N, O and P), GO:0022613, ribonucleoprotein complex biogenesis (clusters F, G and N) and GO:0007507, heart development (clusters M, O and P). The diversity of GO categories to us suggests that multiple systems are differentially expressed during early development in these three charr morphs.

Our published data (Guðbrandsson et al., 2016) had revealed higher expression of genes related to mitochondrial and energy metabolism in aquaculture compared to SB-charr. We hypothesized that this might reflect higher metabolism in the aquaculture charr (due to artificial selection for increased growth) or reduced metabolism in the small benthic charr (adapting to the spring habitat). The current data support the former explanation, because only one GO category functionally related to those processes is significant in our analysis (GO:0022900, electron transport chain) in a cluster were SB does not stand out (cluster K).

In summary, the data revealed considerable expression separation of these three sympatric morphs, during early development. The expression divergence was seen in multiple genes and diverse biological systems. This suggests that the morphs differ in many aspects of development and physiology and that these differences manifest in embryos well prior (100–200 *τs*) to hatching (about 270–280 *τs*).

### Verification of differential expression with qPCR

**Figure 6:**
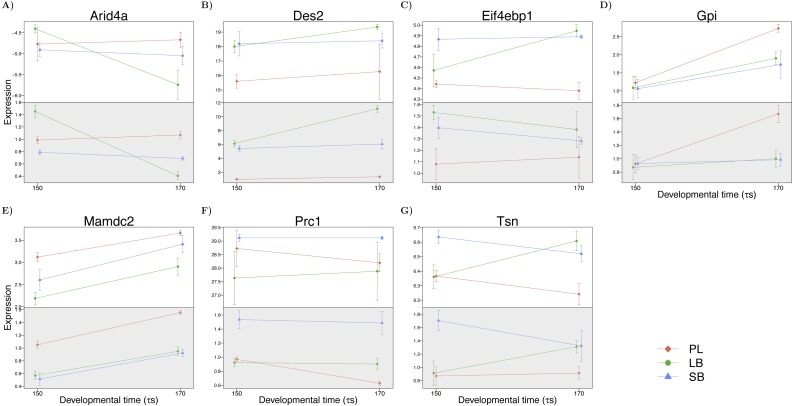
Expression of seven genes that differed between benthic (SB and LB) and limnetic (PL) charr in the transcriptome. (A–G) shows the expression of one gene at developmental timepoints 150 and 170 *τ*s, in the transcriptome (white background) and measured with qPCR (gray background). Colors indicate morph (blue: SB, green: LB, red: PL). The upper panels show expression in transcripts per million (tpm) on log-scale, normalized by the effect of 3′ coverage in the linear model (see Methods). The qPCR expression is normalized to the geometric mean of two reference genes (*actb* and *ub2l3*) and represented relative to one replicate of the PL morph at 150 (*τ*s). Error bars represent 2 standard errors of the mean calculated from three biological replicates each made from a homogenate of three whole embryos.

In order to verify morph specific differences in expression indicated in the data we queried a subset of genes from several of the co-expression clusters with qPCR in whole embryos. We studied the same three morphs (PL-, LB- and SB-charr) and tested seven candidate genes at two developmental timepoints (150 and 170 *τs*) with different expression in the benthic morphs (LB- and SB-charr) and limnetic morph (PL-charr) in the transciptome. Note, the primers amplified mRNA of paralogous genes, which will be less sensitive if the two paralogs differ in expression (as was seen for *natterin-like genes* (Guðbrandsson et al., 2016)). Expression of six genes *MAM Domain Containing 2 (mamdc2)*, *delta(4)-desaturase, sphingolipid 2 (des2)*, *translin (tsn)*, *glucose 6-phosphate isomerase (gpi)*, *protein regulator of cytokinesis 1 (prc1)* and *AT-Rich Interaction Domain 4A (arid4a)*, differed significantly among morphs (*p* < 0.05). The seventh gene *eukaryotic translation initiation factor 4E binding protein 1 (eif4ebp1)* showed a suggestive limnetic and benthic separation in the qPCR (only formally significant at 170 *τs*) (Fig. 6). Notably, *arid4a* showed the same Morph by Time interaction in both the transcriptome and qPCR. In sum, the general agreement between the transcriptome results and the qPCR verification tests on whole embryos, suggests the majority of the ∼2,000 morph effect transcripts represent true differences in expression.

Of those genes, three (*arid4a*, *tsn* and *eif4ebp1*) have known regulatory functions. *Arid4a* encodes a Retinoblastoma binding protein, that has been demonstrated to repress transcription and induce growth arrest in human cell culture (Lai et al., 1999). *Tsn* encodes a protein which positively influences the activity of the RISC complex (Liu et al., 2009). *Eif4ebp1* encodes a repressor of translation initiation, and is a target of *mTOR* (Wang et al., 2005; Dowling et al., 2010). The other genes have diverse functions, *prc1* is a cell cycle related gene (Li, Shridhar & Liu, 2003), *gpi* a glycolytic enzyme differentially expressed in zebrafish development (Lin et al., 2009), *des2* is involved in sphingolypid synthesis (Omae et al., 2004) whereas the function of *mamdc2* is poorly characterized.

In the light of prior data we focus the discussion on the benthic-limnetic patterns of *eif4ebp1* expression. The gene had higher expression in the benthic charr (formally significant in the transcriptome but only the later timepoint with qPCR). Macqueen et al. (2011) found similarly higher expression of this gene and two other *mTOR* pathway related genes in muscles of five small benthic vs two limnetic morphs from south Iceland. Preliminary analyses of this transcriptome (J Gudbrandson et al., 2018, unpublished data) indicate differences in allele frequency of variants in *eif4ebp1* between SB- and PL-charr. These observations do not prove the involvement of *eif4ebp1* in morph differentiation, but call for further study of mTOR pathway genes in different Thingvallavatn morphs and benthic vs. limnetic charr. It must be emphasized that the data presented here are correlative, and do not prove causal influence of these genes on charr development or divergence.

Previously (Ahi et al., 2014; Ahi et al., 2015) we screened for candidate genes involved in craniofacial development, utilizing our published data (Guðbrandsson et al., 2016) and this dataset. We focused on genes with differential expression between limnetic and benthic morphs involved in bone and cartilage development or with craniofacial expression in Zebrafish, and also mined online databases for conserved patterns of co-expression among candidates (Ahi et al., 2014; Ahi et al., 2015). Several genes showed clearly overlapping expression in perichondrial regions of the pharyngeal arches during their formation. Interestingly, binding sites for the transcription factor *ets2*, which shows the same expression pattern, are conserved upstream of the co-expressed genes in species as distantly related as *Oryzias latipes* and *Drosophila melanogaster* (Ahi et al., 2014). A second study revealed more genes with clear benthic-limnetic separation in expression, and pointed to transcription factors in the glucocorticoid and Aryl hydrocarbon pathways as potential modulators of benthic-limnetic diversity (Ahi et al., 2015).

These results and the current data suggest that multiple developmental systems have diverged in these three sympatric morphs, likely reflecting substantial genetic differentiation at multiple loci. Therefore an obvious next step is to ascertain genome-wide data on the genetic separation of the morphs, for instance by mining this transcriptome for sequence polymorphisms (already in progress, J Guðbrandsson et al., 2018, unpublished data). Alternative approaches could be whole genome scans of divergence e.g., (Jones et al., 2012; Halldórsdóttir & Árnason, 2015) or quantitative trait loci (QTL)/association studies e.g., (Zimmerman, Palsson & Gibson, 2000; Palsson et al., 2005) of specific ecological traits to identify putative causative factors and variants that differentiate these sympatric morphs. Furthermore as dwarf charr are found in multiple locations, it would be interesting to study their transcriptomes, perhaps at finer developmental resolution to test the reproducibility of developmental changes in evolution. Also, while the sympatric morphs of Lake Thingvallavatn are clearly demarcated phenotypically, subtler signs of polymorphism are found in several lakes (Woods et al., 2012). Molecular and genetic analyses of several morph pairs varying in degree of divergence would be exciting.

## Conclusions

The differences in trophic morphology, habitat use and life history traits among the sympatric charr morphs in Lake Thingvallavatn have intrigued students of fish biology and evolution for more than a century (Sæmundsson, 1904; Frioriksson, 1939; Snorrason et al., 1989; Skúlason et al., 1996; Ahi et al., 2015). Genes, environment and parental effects are known to contribute to the morph differences (Snorrason et al., 1994; Skúlason, Snorrasson & Jónsson, 1999; Leblanc, Kristjánsson & Skúlason, 2016). The LB-, SB- and PL-charr differ significantly at the genetic level, but the estimates of relatedness and phylogenetic relationships of the three morphs vary by studies (Volpe & Ferguson, 1996; Gíslason, 1998; Kapralova et al., 2011). With the current experimental design parental effects can not be excluded. We still postulate that large fraction of the expression differences between morphs stem from genetic differences. The observed pattern at the expression level, that all morphs are separated and the benthic morphs are more similar (this data), suggests that it is important to follow this work with investigation of the polymorphism trends in the transcriptome (Johannes Guðbrandsson et al., in preparation). A population genomic screen may be needed to evaluate these relationships and the origin of the Lake Thingvallavatn morphs. We find that expression of multiple genes differs between the three charr morphs during early development and prior to hatching. This observation and previous studies on co-expressed genes (Ahi et al., 2014; Ahi et al., 2015) indicate that during development, upstream regulatory mechanisms may be acting differently in these morphs. Thus differential expression of regulators such as *tsn*, *ahr2* (Ahi et al., 2015) or *eif4ebp1* (Macqueen et al., 2011), lead us to speculate that they may influence expression at multiple loci and cause differences in ecologically important traits, e.g., concerning the structure and function of the feeding apparatus and muscle growth (Sandlund et al., 1992; Macqueen et al., 2011).

Although the genes identified here and in our previous studies (Ahi et al., 2014; Ahi et al., 2015) may constitute key links in developmental cascades that through differential expression (timing and pattern) induce morph differences, the underlying genetic differences have not been identified. They may reside in the *cis*-elements of some of these genes, but more likely in up-stream members of pathways that regulate development. Identifying the causative molecular changes associated with evolutionary divergence is not straightforward (Santure et al., 2015), in part because of the pleiotropic nature of metabolic, homeostatic and developmental systems (Paaby & Rockman, 2013). One intriguing question is whether the heritable expression differences between morphs is due to variation in one gene, few genes or many QTLs? Our combined data (Ahi et al., 2014; Ahi et al., 2015; Guðbrandsson et al., 2016) including the present data, argues against a monogenic model, i.e., where a single gene is responsible for the observed morph differences. The data is, in our opinion, more consistent with divergence in multiple systems and thus in many genomic regions among morphs (polygenic model). To disentangle the molecular systems responsible for morph divergence the anatomical focus must be sharpened by studying gene expression in specific tissues (head or jaw) or cell types at particular developmental time-points. Another option is a genomic scan of divergence that may implicate specific loci or systems. The intersection of genes or systems that show both genetic and expression difference between morphs is naturally interesting. Although several studies have found one or few genes that contribute heavily to key traits among closely related morphs/species (Shapiro et al., 2004; Johnston et al., 2013; Kunte et al., 2014) in many cases divergence in numerous genes influencing multiple cellular, developmental and physiological systems is a more likely scenario (Flint & Mackay, 2009; Coolon et al., 2014; Laporte et al., 2015), as seems to be the case for the Arctic charr morphs in Lake Thingvallavatn.

##  Supplemental Information

10.7717/peerj.4345/supp-1 Table S1 Supplementary material Sequencing effort, quality trimming, mapping and estimated insert size for each sample.

10.7717/peerj.4345/supp-2 Table S2 Results from differential expression analysis. Multiple testing corrected *p*-values (*q*-values) and overall log fold change between morphs from the full model for each transcript are shown (Model FM in methods). **TransID:**Trinity transcript identification code
**q_MxT:** *Q*-value for the Morph × Time interaction term from likelihood ratio test between model FM and R1 (see methods)
**q_Morph:** *Q*-value for the Morph term from likelihood ratio test between model R1 and R2 (see methods)
**q_Time:** *Q*-value for the Time term from likelihood ratio test between model R1 and R3 (see methods)
**q_Tprime:** *Q*-value for the 3’-bias terms from likelihood ratio test between model FM and R4 (see methods)
**FC_PL_SB:** Log fold change between PL and SB. Parameters extract from the full model (FM)
**FC_PL_LB:** Log fold change between PL and LB. Parameters extract from the full model (FM)
**FC_LB_SB:** Log fold change between LB and SB. Parameters extract from the full model (FM)
**Cluster:** Expression cluster for transcripts with significant Morph or Morph ×Time interaction
**SSncbi_Top_BLASTN_gene_name:** Gene name based on top blastn hit in the NCBI *Salmo salar* Annotation
**SalmoBase_Top_BLASTN_gene:** Id for top blastn gene in SalmoBase *Salmo salar* Annotation

10.7717/peerj.4345/supp-3 Table S3 The results of gene ontology analyses of the transcripts with significant expression difference between morphs (or morph by time interaction) in the Arctic charr developmental transcriptome. The enrichment was tested for transcripts and genes (SalmoBase) **GO.ID:** Identification number for Gene Ontology categories
**Term:** The Gene Ontology term or description of the category
**numDE.t:** Number of transcripts within expression cluster in each GO-category
**numIn.t:** Total number of transcripts in each GO-category
**fdr.t:** Multiple testing corrected P-value (FDR) for enrichment based on transcripts
**p.t:** Uncorrected P-value for enrichment based on transcripts
**numDE.g:** Number of genes (SalmoBase) within expression cluster in each GO-category
**numIn.g:** Total number of genes (SalmoBase) in each GO-category
**fdr.g:** Multiple testing corrected P-value (FDR) for enrichment based on genes
**p.g:** Uncorrected P-value for enrichment based on genes
**Cluster:** Expression cluster
**GOclust:** Super-GO-categories based on categories semantic similarity (see Methods)

10.7717/peerj.4345/supp-4 Table S4 Information about genes used in qPCR. Detailed gene names, primer sequence, amplicon size and transcripts in the assembly used for comparison **Gene Symbol:** The symbol or short gene name used in figures and text
**Description:** Full name of each gene
**Forward primer:**Sequence for the forward qPCR primer in 5’-3’ orientation
**Reverse primer:** Sequence for the reverse qPCR primer in 5’-3’ orientation
**Amplicon size:** Size of the sequence amplified in the qPCR reaction
**Transcripts:** The id of assembled transcripts in the transciptome that the primers bind to and were used for comparison of expression. If there are more than one transcipts for each gene the ids are separated by a semicolon.

10.7717/peerj.4345/supp-5 Figure S1 Developmental events in the LB-charr at relative ages 100–200*τ*s (A) Developmental events in the LB-charr at relative ages 100–200*τ*s (dorsal views of 6 time points). By 100*τ*s heart contractions have begun and second gill fissures have started to form. By 140*τ*s all somites are formed and eye pigmentation has started to appear. Between 150–200*τ*s the upper and lower jaws separate from the yolk, the first melanophores appear and start spreading from the head along the trunk and the operculum covers the first gill arch. Scale bar: 1 mm. (B) Development and growth of craniofacial cartilage elements at pre-hatching stages at relative stages 140, 150, 160, 170 and 200*τ*s LB-charr embryos (ventral views of 5 time points): no craniofacial elements are seen at 140*τ*s; at 150*τ*s the trabeculae, Meckel’s cartilages, and palatoquandrates can be seen clearly; at 160*τ*s the hyoid arch and the ceratobranchials (cb) 1–3 become visible; at 170*τ*s: basibranchial (bb) cartilages and cb 1–4 have emerged; at 200*τ*s the fusing of the ethmoid plate has started and the hypohyal (hh), hypobranchial cartilages (hb) 1–2 and cb 1–5 are visible. Scale bar: 1 mm.

10.7717/peerj.4345/supp-6 Figure S2 Principal component analysis (PCA) of expression data for all transcripts (A) shows the first and second PCA-axis and (B) the third and fourth PCA-axis. The first PCA-axis correlates with developmental time. Samples from 2011 (SB100, SB140 and PL140) do not deviate largely from other samples for any of the PCA-axis. Standardized expression normalized by 3′-coverage was used as input. Samples are colored according to morph and time, and sample labels are shown for each replicate.

10.7717/peerj.4345/supp-7 Figure S3 Effect of 3’-bias correction on the number of transcripts differently expressed by developmental timepoint (time), morph and interaction of morph and time (int) Each figure shows the intersection size (upper barplot) - the number of transcripts significant for each one or a combination of two or more factors (indicated by dots), while the set size barplot (lower) shows cumulated number for each factor. Indicated are the number transcripts differently expressed (DE) with (“effect”) and without (“effect3”) taking 3’-bias into account. For example, the Morph category in figure B represents the number of DE-transcripts when 3’-bias is taken into account, but Morph3 category denotes transcripts that are DE when 3′-bias estimator was dropped from the model. The different panels represent the impact of 3’-bias on (A) the Time and Morph by Time interaction (int) terms, (B) the Morph term, (C) the Time term in isolation and (D) only the M×T interaction (int) term. The dots indicate the significant factors or their combinations.

## Additional Information and Declarations

### Field Study Permissions

The following information was supplied relating to field study approvals (i.e., approving body and any reference numbers): Fishing was performed with the permissions both from the owner of the land in Mjóanes and from the Thingvellir National Park commission.

### DNA Deposition

The following information was supplied regarding the deposition of DNA sequences: The sequencing reads from the 48 samples were deposited into the NCBI SRA archive under BioProject identifier PRJNA391695 and with accession numbers: SRS2316381 to SRS2316428.

### Data Availability

The following information was supplied regarding data availability: Table S5: Guðbrandsson, Jóhannes (2018): Annotation of de novo assembly from Arctic charr in Lake Thingvallavatn, Iceland. figshare. https://doi.org/10.6084/m9.figshare.5445610.v2.
