# Supplementary material for: Differential gene expression during early development in recently evolved and sympatric Arctic charr morphs
Source: PeerJ. 2018 Feb 7;6:e4345. doi: 10.7717/peerj.4345 (PMC5807978; doi:10.7717/peerj.4345)
Supplement: Table S1 — Sequencing effort, quality trimming, mapping and estimated insert size for each sample. [file peerj-06-4345-s001.pdf]

Table S1. Sequencing effort, quality trimming, mapping and estimated insert size for each sample.

Morph	Relative age	Rep	Index	Lane	Nr of reads	After trim[a]	KaAP[b]	KaFL [c]	RIN
LB	140	A	CCGTCC	L004	27,701,961	0.997	0.777	205.77	9.8
LB	140	B	AGTTCC	L003	14,573,957	0.996	0.795	186.08	9.7
LB	140	C	ATGTCA	L002	23,771,531	0.996	0.798	190.16	9.8
LB	150	A	GTGAAA	L008	31,473,035	0.996	0.786	215.33	9.5
LB	150	B	AGTTCC	L006	20,016,988	0.998	0.808	178.49	9.3
LB	150	C	ATGTCA	L005	30,253,324	0.996	0.814	183.35	9.2
LB	160	A	GTGAAA	L005	14,981,564	0.996	0.811	195.50	8.2
LB	160	B	AGTCAA	L001	6,613,627	0.995	0.801	183.29	8.1
LB	160	C	GTCCGC	L003	4,498,403	0.997	0.817	188.57	7.4
LB	170	A	AGTCAA	L007	28,634,115	0.996	0.808	188.17	9.8
LB	170	B	GTCCGC	L006	54,668,356	0.998	0.809	182.10	10.0
LB	170	C	AGTCAA	L004	22,356,753	0.996	0.810	188.93	9.9
LB	200	A	ATGTCA	L008	16,701,328	0.997	0.791	173.07	9.5
LB	200	B	GTGAAA	L002	8,978,602	0.996	0.777	205.14	9.9
LB	200	C	CCGTCC	L001	20,497,625	0.998	0.763	208.80	9.6
PL	100	A	AGTCAA	L008	16,674,885	0.996	0.798	195.50	10.0
PL	100	B	GTCCGC	L007	9,288,679	0.997	0.798	190.52	10.0
PL	100	C	CCGTCC	L002	40,970,653	0.997	0.787	213.78	10.0
PL	140	A	GTCCGC	L004	37,166,151	0.996	0.799	189.61	9.5
PL	140	B	ATGTCA	L003	6,519,881	0.996	0.802	180.05	9.7
PL	140	C	AGTCAA	L002	26,836,537	0.997	0.786	202.68	9.7
PL	150	A	CCGTCC	L008	27,515,495	0.998	0.779	219.85	9.9
PL	150	B	ATGTCA	L006	21,572,729	0.998	0.807	180.36	9.8
PL	150	C	AGTCAA	L005	31,276,989	0.996	0.809	191.31	9.9
PL	160	A	CCGTCC	L005	19,719,655	0.997	0.789	207.33	8.4
PL	160	B	AGTTCC	L001	46,868,956	0.998	0.795	190.61	9.5
PL	160	C	GTGAAA	L003	19,583,357	0.995	0.788	197.48	8.7
PL	170	A	AGTTCC	L007	43,829,383	0.996	0.800	183.36	9.5
PL	170	B	GTGAAA	L006	30,612,275	0.997	0.797	189.97	9.3
PL	170	C	AGTTCC	L004	13,537,568	0.996	0.797	195.55	9.8
SB	100	A	AGTTCC	L008	20,853,072	0.996	0.805	189.29	10.0
SB	100	B	GTGAAA	L007	11,073,164	0.996	0.804	188.17	9.9
SB	100	C	GTCCGC	L001	19,435,986	0.998	0.806	192.59	9.5
SB	140	A	GTGAAA	L004	11,034,246	0.995	0.802	199.26	9.1
SB	140	B	AGTCAA	L003	35,722,829	0.996	0.802	189.73	9.8
SB	140	C	AGTTCC	L002	21,053,359	0.997	0.801	196.91	9.9
SB	150	A	GTCCGC	L008	19,505,065	0.997	0.794	209.40	-
SB	150	B	AGTCAA	L006	44,039,656	0.998	0.814	169.16	9.9
SB	150	C	AGTTCC	L005	17,412,112	0.996	0.789	195.52	10.0
SB	160	A	GTCCGC	L005	30,431,301	0.997	0.805	198.43	10.0
SB	160	B	ATGTCA	L001	28,770,693	0.997	0.796	186.67	10.0
SB	160	C	CCGTCC	L003	28,085,159	0.997	0.789	199.52	9.8
SB	170	A	ATGTCA	L007	31,585,623	0.996	0.797	199.35	10.0
SB	170	B	CCGTCC	L006	17,485,747	0.999	0.793	191.56	10.0
SB	170	C	ATGTCA	L004	20,881,241	0.996	0.810	184.47	10.0
SB	200	A	CCGTCC	L007	14,769,479	0.998	0.805	194.85	9.8
SB	200	B	GTCCGC	L002	11,357,151	0.997	0.793	202.11	9.6
SB	200	C	GTGAAA	L001	86,894,303	0.997	0.769	196.70	9.6
			Average		24,751,761	0.997	0.797	193.42	9.58
			Min		4,498,403	0.995	0.763	169.16	7.4
			Max		86,894,303	0.999	0.817	219.85	10.0

[a] The proportion of reads retained as pairs after trimming
[b] The proportion of raw reads aligned with kallisto
[c] Estimated fragment length by kallisto
