# Supplementary figures and images for: Differential gene expression during early development in recently evolved and sympatric Arctic charr morphs
Source: PeerJ. 2018 Feb 7;6:e4345. doi: 10.7717/peerj.4345 (PMC5807978; doi:10.7717/peerj.4345)

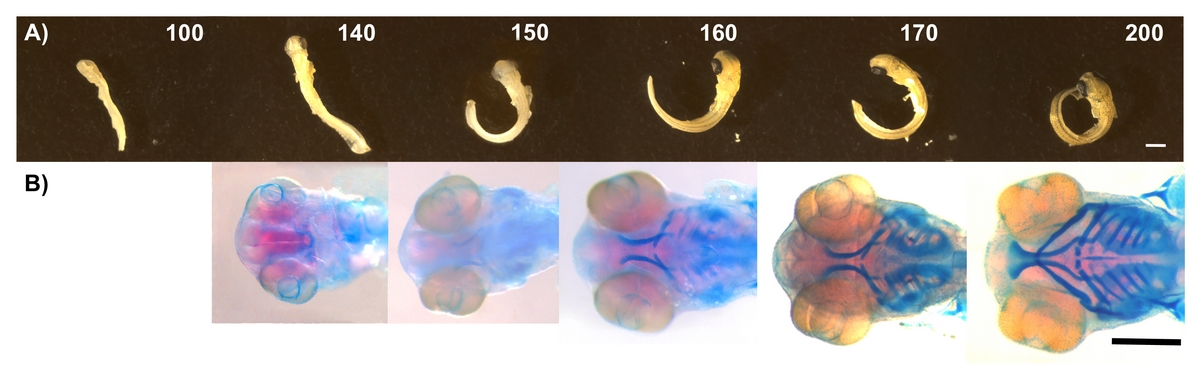

Supplement: Figure S1 — (A) Developmental events in the LB-charr at relative ages 100–200τs (dorsal views of 6 time points). By 100τs heart contractions have begun and second gill fissures have started to form. By 140τs all somites are formed and eye pigmentation has started to appear. Between 150–200τs the upper and lower jaws separate from the yolk, the first melanophores appear and start spreading from the head along the trunk and the operculum covers the first gill arch. Scale bar: 1 mm. (B) Development and growth of craniofacial cartilage elements at pre-hatching stages at relative stages 140, 150, 160, 170 and 200τs LB-charr embryos (ventral views of 5 time points): no craniofacial elements are seen at 140τs; at 150τs the trabeculae, Meckel’s cartilages, and palatoquandrates can be seen clearly; at 160τs the hyoid arch and the ceratobranchials (cb) 1–3 become visible; at 170τs: basibranchial (bb) cartilages and cb 1–4 have emerged; at 200τs the fusing of the ethmoid plate has started and the hypohyal (hh), hypobranchial cartilages (hb) 1–2 and cb 1–5 are visible. Scale bar: 1 mm. [file peerj-06-4345-s005.jpg]

A)

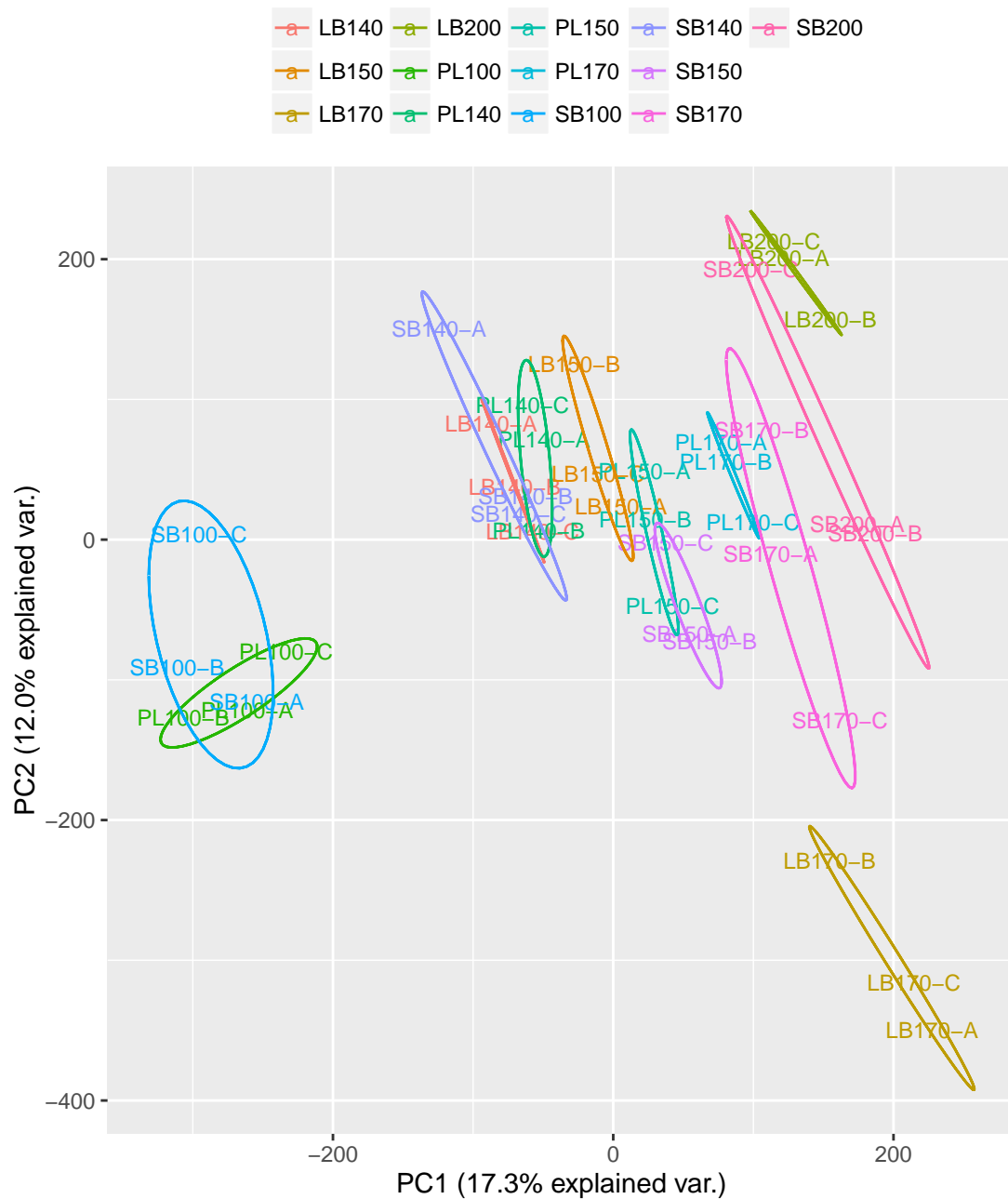

B)

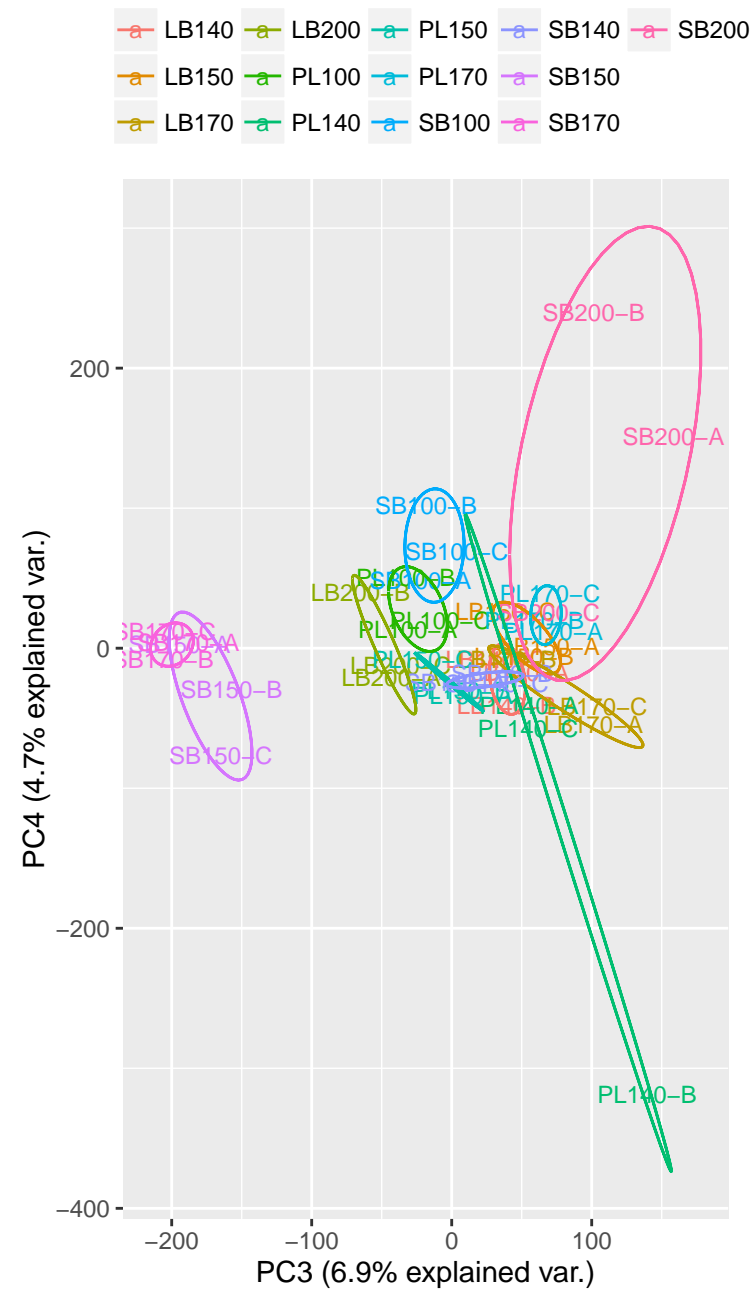

Supplement: Figure S2 — (A) shows the first and second PCA-axis and (B) the third and fourth PCA-axis. The first PCA-axis correlates with developmental time. Samples from 2011 (SB100, SB140 and PL140) do not deviate largely from other samples for any of the PCA-axis. Standardized expression normalized by 3′-coverage was used as input. Samples are colored according to morph and time, and sample labels are shown for each replicate. [file peerj-06-4345-s006.pdf]

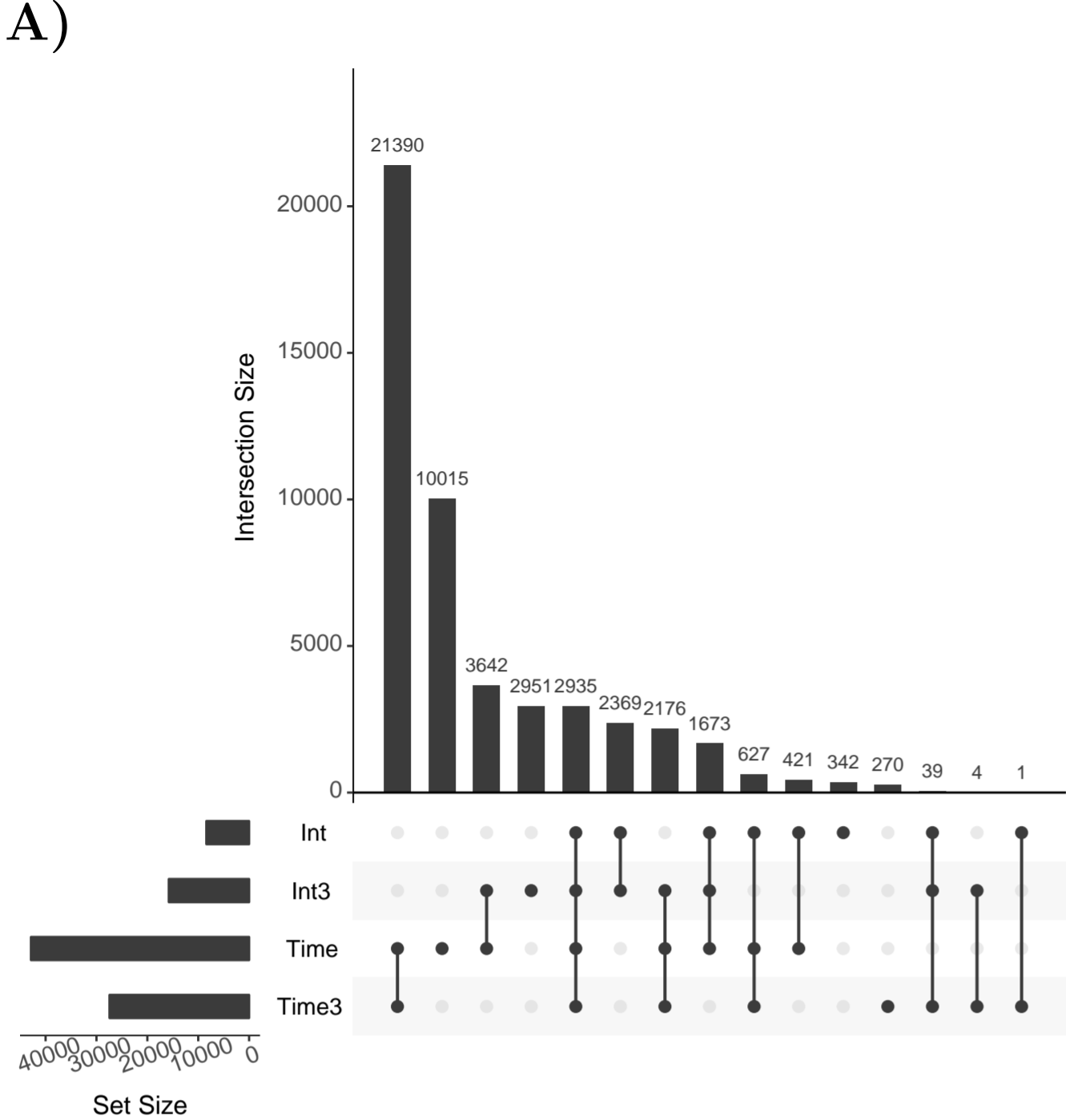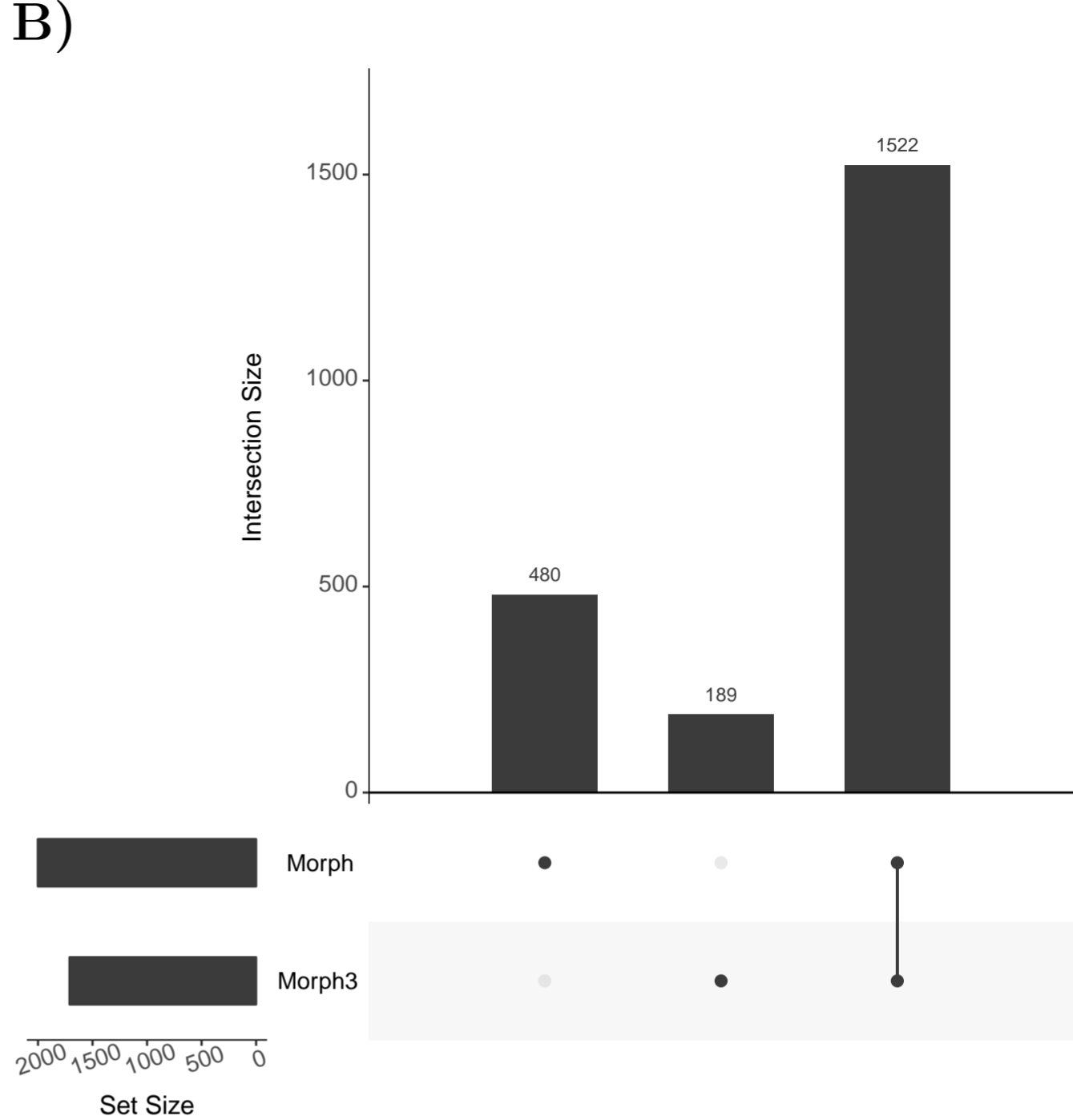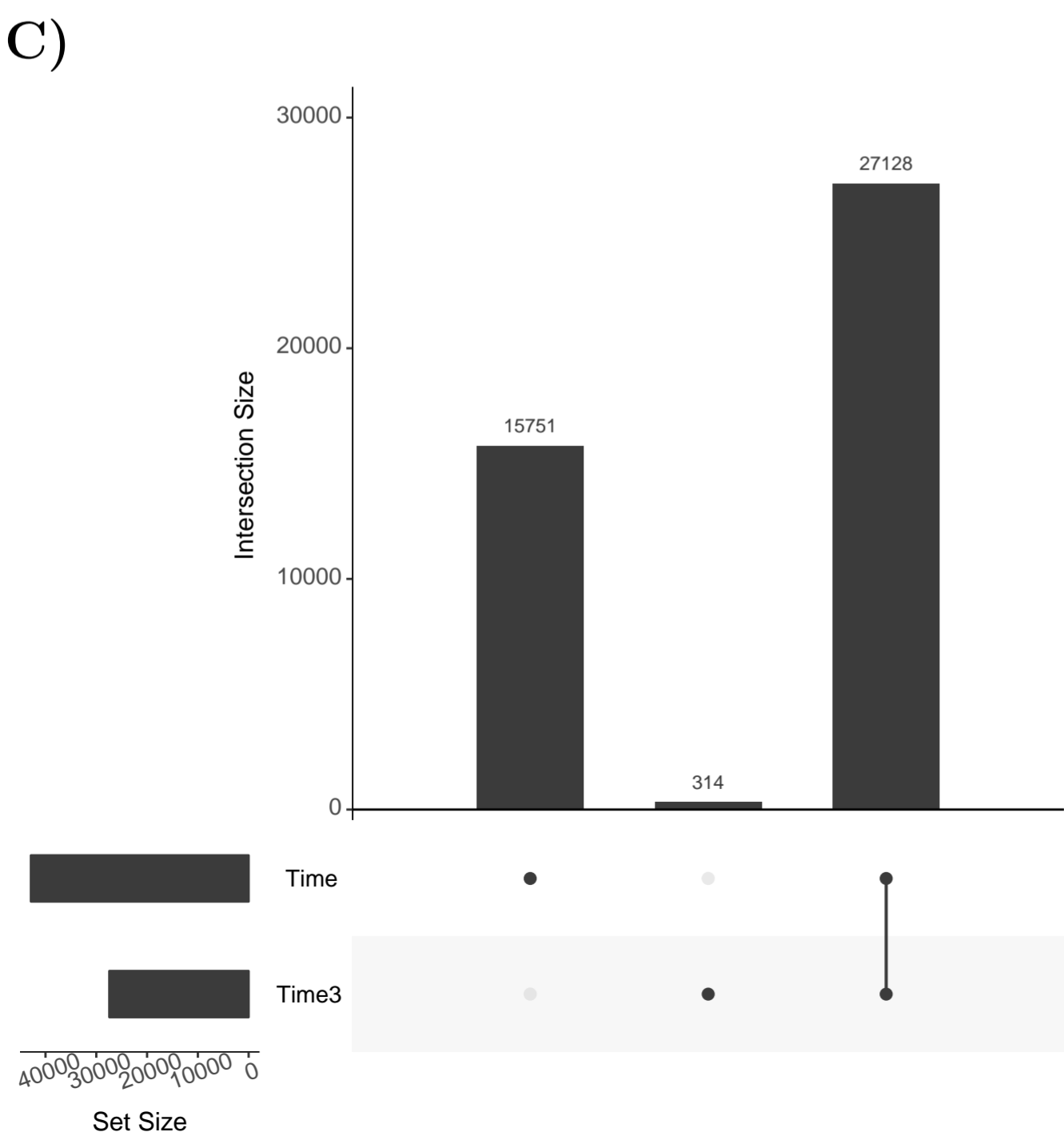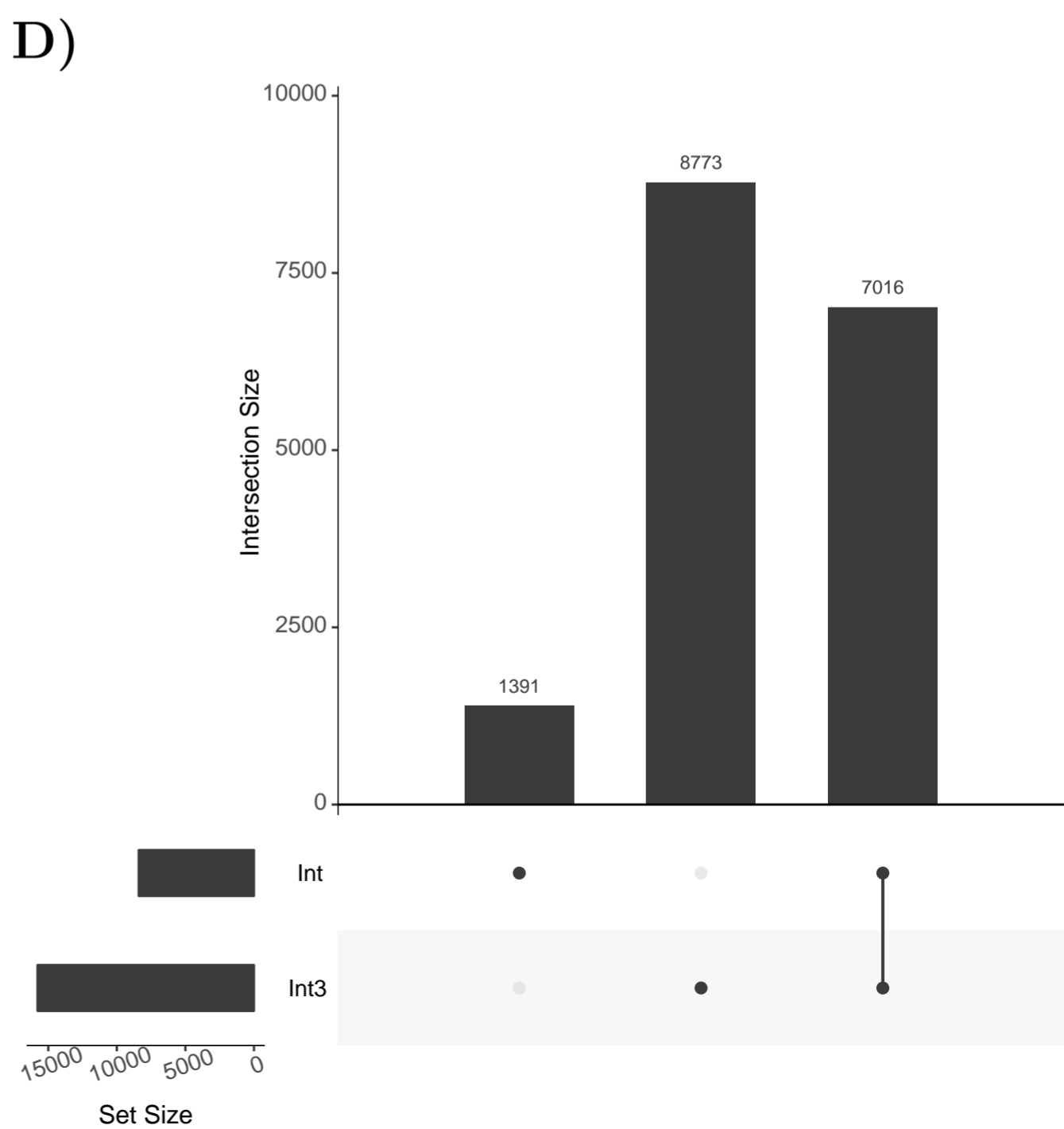

Supplement: Figure S3 — Each figure shows the intersection size (upper barplot) - the number of transcripts significant for each one or a combination of two or more factors (indicated by dots), while the set size barplot (lower) shows cumulated number for each factor. Indicated are the number transcripts differently expressed (DE) with (“effect”) and without (“effect3”) taking 3’-bias into account. For example, the Morph category in figure B represents the number of DE-transcripts when 3’-bias is taken into account, but Morph3 category denotes transcripts that are DE when 3′-bias estimator was dropped from the model. The different panels represent the impact of 3’-bias on (A) the Time and Morph by Time interaction (int) terms, (B) the Morph term, (C) the Time term in isolation and (D) only the M×T interaction (int) term. The dots indicate the significant factors or their combinations. [file peerj-06-4345-s007.pdf]
